# Recurrent patterns of microdiversity in a temperate coastal marine environment

**DOI:** 10.1038/ismej.2017.165

**Published:** 2017-10-24

**Authors:** Meghan Chafee, Antonio Fernàndez-Guerra, Pier Luigi Buttigieg, Gunnar Gerdts, A Murat Eren, Hanno Teeling, Rudolf I Amann

**Affiliations:** 1 Max Planck Institute for Marine Microbiology, Bremen, Germany; 2 Jacobs University Bremen gGmbH, Bremen, Germany; 3 University of Oxford, Oxford e-Research Centre, Oxford, UK; 4 HGF-MPG Bridge-Group for Deep Sea Ecology and Technology, Alfred-Wegener Institute, Helmholtz-Zentrum für Polar- und Meeresforschung, Bremerhaven, Germany; 5 Alfred-Wegener Institut, Helmholtz-Zentrum für Polar- und Meeresforschung, Biologische Anstalt Helgoland, Helgoland, Germany; 6 University of Chicago, Department of Medicine, Knapp Center for Biomedical Discovery, Chicago, IL, USA; 7 Marine Biological Laboratory, Woods Hole, MA, USA

## Abstract

Temperate coastal marine environments are replete with complex biotic and abiotic interactions that are amplified during spring and summer phytoplankton blooms. During these events, heterotrophic bacterioplankton respond to successional releases of dissolved organic matter as algal cells are lysed. Annual seasonal shifts in the community composition of free-living bacterioplankton follow broadly predictable patterns, but whether similar communities respond each year to bloom disturbance events remains unknown owing to a lack of data sets, employing high-frequency sampling over multiple years. We capture the fine-scale microdiversity of these events with weekly sampling using a high-resolution method to discriminate 16S ribosomal RNA gene amplicons that are >99% identical. Furthermore, we used 2 complete years of data to facilitate identification of recurrent sub-networks of co-varying microbes. We demonstrate that despite inter-annual variation in phytoplankton blooms and despite the dynamism of a coastal–oceanic transition zone, patterns of microdiversity are recurrent during both bloom and non-bloom conditions. Sub-networks of co-occurring microbes identified reveal that correlation structures between community members appear quite stable in a seasonally driven response to oligotrophic and eutrophic conditions.

## Introduction

Microbes in coastal marine environments are influenced by multiple biotic and abiotic elements that mediate relationships between community members. Shifts in community structure can be modulated by changes in bottom-up substrate availability induced by seasonal forcing such as solar angle and upwelling and by top–down predator–prey interactions (reviewed in Fuhrman *et al.*, 2015). These dynamics are amplified in temperate coastal marine zones when phytoplankton form massive blooms each spring and summer. Following winter, increases in light and temperature favor algal growth in spring. Upon bloom termination, algae release a complex array of dissolved organic matter. These organic substrates are almost exclusively accessible by heterotrophic bacterioplankton as part of the microbial loop (Azam *et al.*, 1983; Azam and Ammerman, 1984; Ducklow and Carlson, 1992). Many of the bacterioplankton responding to spring bloom events are represented by fast-growing copiotrophs, triggering successions of specialized clades that are able to rapidly incorporate algal-derived dissolved organic matter into their biomass using diverse repertoires of specialized transporters and carbohydrate-active enzymes (Thomas *et al.*, 2011; Koropatkin *et al.*, 2012; Teeling *et al.*, 2012, 2016; Buchan *et al.*, 2014; Cuskin *et al.*, 2015; Xing *et al.*, 2015). In turn, these organisms constitute a new pool of particulate bacterial biomass, which is available to protozoa and other higher trophic levels (Azam *et al.*, 1983). Production that is not catabolized is typically exported to sediments below (Legendre and Le Fèvre, 1989).

Biodiversity studies at long-term sampling stations have revealed broadly recurrent patterns within marine planktonic communities (Steinberg *et al.*, 2001; Gilbert *et al.*, 2012; Chow *et al.*, 2013; Fuhrman *et al.*, 2015). However, many of these studies employ monthly sampling intervals that do not adequately capture community dynamics during productive bloom events, which can elicit rapid shifts in community structure on the order of weeks (Ward *et al.*, 2017), or days (Needham and Fuhrman, 2016). Annually replicated studies employing high-frequency sampling throughout both baseline and bloom periods are required to discern stable forces that initiate, regulate and maintain successional changes in microbial communities, to examine the extent of recurrence across years, and to identify non-random co-occurrences between microorganisms that can be used to formulate hypotheses about ecologically relevant *in situ* community interactions.

Although previous multi-year studies have identified seasonally repeating patterns of marine microbial diversity based on 16S ribosomal RNA (rRNA) surveys, in many such studies phylogenetic characterization has remained coarsely defined (Fuhrman *et al.*, 2006; Gilbert *et al.*, 2009; Teeling *et al.*, 2016). Microbial clades tend to maintain broadly conserved ecological characteristics (Philippot *et al.*, 2010; Martiny *et al.*, 2015), and in some systems, coarser levels of taxonomy show improved correlations with environmental drivers (Lu *et al.*, 2016). However, it has been shown in marine systems that sub-operational taxonomic unit (OTU) level patterns of microdiversity reveal meaningful variation in response to environmental variables (Eren *et al.*, 2013; Ward *et al.*, 2017) and can display stronger patterns of correlation when used to disentangle highly specific interactions, like those between microbial hosts and their viruses (Needham *et al.*, 2017). High phylogenetic resolution is thus critical to identify relationships and potential interactions between community members as they respond to seasonal fluctuations in environmental variables.

The long-term research site Helgoland Roads (German Bight, North Sea) hosts annual spring and summer phytoplankton blooms. In an effort to improve the resolution of community dynamics during both winter and bloom conditions, we investigated the free-living bacterioplankton community inhabiting Helgoland Roads surface waters using weekly sampling intervals across 3 complete years. In order to improve the resolution of the community diversity, we characterized patterns of microdiversity using Minimum Entropy Decomposition (MED) to cluster 16S rRNA gene amplicons. Using these highly resolved taxonomic units in combination with network analysis, we aimed to identify and characterize recurrent modules of co-varying microbes.

## Materials and methods

### Sampling site

The long-term Helgoland Roads sampling site is adjacent to Helgoland Island, which lies ~60 km offshore in the southeastern North Sea in the German Bight. This site represents a coastal–oceanic transition zone (Raabe and Wiltshire, 2009) with westerly currents transporting marine water from the English Channel and the rivers Elbe and Weser, providing periodic incursions of freshwater (Wiltshire *et al.*, 2009). Samples were taken at the station 'Kabeltonne' (54° 11' 17.88'' N, 7° 54' 0'' E) from 2010 to 2012, which is located between Helgoland Island and the minor island, Düne, where water depths fluctuate from 7 to 10 m over the tidal cycle. Long-term physicochemical and plankton data have been monitored continuously since 1962 as part of the Helgoland Roads time series and are accessible via the Open Access Library Pangaea (http://www.pangaea.de).

### Physicochemical, phytoplankton and bacterioplankton sampling

Physicochemical data collected includes dissolved inorganic nitrogen (NO_2_^−^+NO_3_^−^+NH_4_^+^), silicate (SiO_2_), phosphate (PO_4_^3−^), salinity, water temperature, chlorophyll *a* (chl *a*) and Secchi disk depth (Supplementary Table S1). Counts of major phytoplankton groups were obtained through microscopic morphological identification (Supplementary Table S2), as described previously (Wiltshire *et al.*, 2009), and include diatoms (pennales and centrales), dinoflagellates, flagellates, coccolithophorids, ciliates, *Phaeocystis* spp. and *Chattonella* spp.

Samples of 2010–2012 free-living archaeal and bacterial picoplankton (collectively termed ‘bacterioplankton’) were taken at the station 'Kabeltonne' at bi-weekly to weekly intervals, as described previously (Teeling *et al.*, 2016). In brief, surface seawater samples (ca. −1 m) were pre-filtered at 10 μm, followed by removal of the particle-attached fraction at 3 μm with final collection of the free-living bacterioplankton fraction on 0.2 μm filters. In total, there were 142 free-living samples: 30 in 2010, 68 in 2011 and 44 in 2012. Sampling in 2010 is patchy during winter and late spring, however, 2011 and 2012 are represented by evenly spaced samples. Annual DAPI total cell counts of bacterioplankton abundance were available from a previous study in 2009 (Teeling *et al.*, 2012) and 2010 (Supplementary Table S3).

### V4 16S rRNA gene sequencing

DNA was extracted from the free-living 0.2–3 μm fraction and sequenced by the Department of Energy Joint Genome Institute (DOE-JGI, Walnut Creek, CA, USA) using V4 region of the 16S rRNA gene using primers 515 F (5′-GTGCCAGCMGCCGCGGTAA-3′) and 806 R (5′-GGACTACHVGGGTWTCTAAT-3′) and Illumina MiSeq 2 × 250 bp chemistry as described previously (Lucas *et al.*, 2015). 16S rRNA gene sequences are available from the DOE-JGI website GOLD database (Project ID: Gp0056779) as part of the community sequencing project COGITO. As a note, the V4 16S rRNA gene primers used in this study do not include primer revisions outlined by Apprill *et al.*, (2015), which better cover the alphaproteobacterial clade SAR11; SAR11 abundances in our data set are thus expected to be significantly under-represented, whereas *Gammaproteobacteria* might be over-represented (Parada *et al.*, 2016).

### Minimum Entropy Decomposition

Raw paired-end reads were merged and quality filtered using Eren *et al.* (2013) to retain pairs with no mismatches in the overlapping regions, followed by primer trimming with cutadapt (Martin, 2011) with a final average length of 253 bp. Sequences were clustered using Minimum Entropy Decomposition (MED) (Eren *et al.*, 2015). MED applies the principle of oligotyping (Eren *et al.*, 2013), which uses Shannon entropy to iteratively partition amplicons at single nucleotide resolution, often providing more accurate descriptions of closely related but distinct taxa (that is, taxa with >97% sequence identity; Utter *et al.*, 2016). We thus differentiate these MED clusters from conventional OTUs by referring to them as ‘oligotypes’. During MED, we used a minimum substantive abundance (-M) of 100 to filter low-abundant oligotypes with the decomposition of one nucleotide position at a time (-d 1). These settings removed most singletons and doubletons, affecting alpha diversity measures of absolute richness. In total, after merging and quality filtering, 15 014 510 high-quality tags were obtained for MED clustering. A total of 4271 representative nodes were decomposed from the 3-year data set representing 13 259 298 sequences (average 93 375 sequences per sample). The remaining 1 755 212 sequences were identified as outliers by MED owing to filtering by either maximum variation cutoff, as determined intrinsically by MED through mean read length, or by falling below the minimum substantive abundance.

Representative oligotypes were classified against the SILVA v123 database. The following threshold was used to determine a database match: (sequence identity+alignment coverage)/2⩾93%, with the remainder classified as having no relative. In total, 77 of the 4271 OTUs did not contain a database match. Half of these ‘no relatives’ had low score hits to chloroplast and mitochondria. All 404 OTUs classified as chloroplast, mitochondria or ‘no relative’ were removed before downstream analysis (3 867 remaining OTUs). SILVA v123 includes major changes in taxonomy for abundant North Sea clades, particularly for *Roseobacter-*related lineages including *Planktomarina* (formerly DC5-80-3 lineage), *Amylibacter* (formerly NAC11-7 lineage) and *Ascidiaceihabitans* (formerly *Roseobacter* OCT lineage). The final abundance matrix includes oligotype ids and SILVA genus-level classifications (Supplementary Table S4). We take proportional read frequencies as a proxy for community relative abundance.

### Alpha diversity analysis

Rarefaction (interpolation) and prediction (extrapolation) of Hill number sampling curves for species richness, exponential Shannon diversity and inverse Simpson diversity were performed with the R package iNEXT (v 2.0.9) (Chao *et al.*, 2014; Hsieh *et al.*, 2016). Hill numbers express diversity in units of *effective numbers of species:* the number of equally abundant species required to give the same value of a diversity measure. The extrapolation end point was set to twice the mean sample size following removal of samples below the 25th percentile in total sequences. Confidence intervals were calculated using the default 50 bootstrap replicates. We calculated Pielou’s J evenness using the equation *J*=*H*/log(S) where *J*=Pielou’s evenness, *H*=the asymptotic estimated exponential Shannon diversity calculated in iNEXT and *S*=total number of species.

Oligotype and sample data matrices were managed with the R package phyloseq (v 1.14.0) (McMurdie and Holmes, 2013) and plots were made with ggplot2 (v 2.1.0) (Wickham, 2009). Pairwise sequence identity between closely related oligotypes was calculated in Geneious (v8.0) (Kearse *et al.*, 2012) using percent identical sites. A custom R function called ‘taxplot_grep’ was used to generate the bar plots presented in this paper and is available within the script https://github.com/mc68462/medNS/blob/v0.3/taxplot_functions.R. Using a simple regular expression search of taxonomy, users can explore oligotypes and lineages of interest.

### Patterns of oligotype prevalence

We plotted each oligotype in an abundance vs prevalence plot, as in (Barberán *et al.*, 2012), to determine whether oligotypes grouped based on how often they are found across time points. Our measure of prevalence constitutes the percent of samples in which an oligotype appears >500 times, thus filtering out oligotypes present in very low abundance across many samples. The threshold of 500 sequences corresponds to 0.32–1.5% oligotype relative abundance across our 142 samples. We identified oligotypes as being broadly prevalent if found in >75% of samples versus those oligotypes narrowly prevalent in <10% of samples. R code is available at https://github.com/mc68462/medNS/blob/v0.3/oligotype_prevalence.R.

### Network analysis

#### Network construction with SparCC

The aim of our network analysis was to determine whether we could identify recurrent modules of co-varying microbes. Owing to large gaps between sampling time points during winter and late spring (Supplementary Figure S1), we omitted 2010 data from network analysis. Separate unfiltered abundance matrices were extracted for 2011 and 2012 and contained 4250 and 4214 oligotypes, respectively. We performed three filtration steps prior to network construction independent of those described above for our analysis of oligotype prevalence: (1) removal of chloroplast and mitochondria oligotypes, (2) removal of oligotypes present in <10% of samples and (3) removal of oligotypes present only in a single year through an intersection of 2011 and 2012 data sets. In step 1 filtration of chloroplasts and mitochondria, we removed 404 and 391 oligotypes (6.4 and 6.2% of total amplicon abundance) in 2011 and 2012 data sets, respectively. In step 2 filtration of low prevalence oligotypes, we removed an additional 85 and 105 oligotypes (0.1% and 0.1% of total amplicon abundance), respectively; low prevalence oligotypes removed were thus present in very low abundance. In the step 3 intersection of 2011 and 2012 data sets, we removed 97 and 54 total oligotypes (0.5 and 0.2% of total amplicon abundance) respectively; oligotypes unique to a single year were thus also present in very low abundance. We performed step 2 filtration to remove rare oligotypes, which might introduce artifacts in the network inference (Berry and Widder, 2014). We performed step 3 filtration because the downstream identification of consensus network modules required the same set of oligotypes to be used in both 2011 and 2012 networks. A total of 3664 oligotypes were retained for the construction of 2011 and 2012 networks. R code is available at https://github.com/mc68462/medNS/blob/v0.3/pre_processing.R

Following filtration, we constructed two independent networks for 2011 and 2012 using Sparse Correlations for Compositional data (SparCC) Friedman and Alm, 2012). SparCC first performs a log-ratio transformation of relative abundance. With compositional data like 16S rRNA genes, abundances are relative (that is, sum to 1); these proportions are thus not independent and do not capture the true correlations underlying absolute abundances (Pawlowsky-Glahn and Buccianti, 2011; Friedman and Alm, 2012). For example, a large increase in the abundance of one oligotype during a period of low diversity necessarily affects the relative abundance of all other oligotypes resulting in correlations (often negative) that reflect the compositional nature of the data, and not the underlying biological processes (Aitchison, 2003; Friedman and Alm, 2012). In log-ratio transformed data the ratio of the proportions of two oligotypes is independent of which other oligotypes are included in the analysis, and thus maintain ‘subcompositional coherence’. We first implemented *SparCC.py*, which performs a log-ratio transformation and then estimates compositionality-robust Pearson correlations between all pairs of oligotypes using the average of 10 iterations sampled from the Dirichlet distribution. We then performed 100 bootstraps using *MakeBootstraps.py* and re-ran *SparCC.py* with 10 iterations to be used in the calculation of two-sided pseudo-*P-*values. The resulting networks are comprised of oligotypes represented as vertices and SparCC correlations represented as undirected weighted edges.

#### Consensus module detection with WGCNA

Following network construction with SparCC, we constructed a topological overlap matrix (TOM) using scripts from the WGCNA R package (Langfelder and Horvath, 2008) as the first step to identify modules of co-varying oligotypes conserved across both 2011 and 2012 SparCC networks (termed consensus modules). As SparCC correlations fall between −1 and 1 and TOM analysis requires values between 0 and 1, we converted the correlations using: 0.5*(cor+1). We performed *TOMsimilarity* for each network using TOMType=’unsigned’ and then performed a scaling step as in Langfelder and Horvath (2016). In the output TOM matrix, a value of 1 means two nodes are (1) connected and (2) share the same neighbors. A TOM value of 0 means two nodes are unconnected and share no neighbors. We then calculated a consensus TOM from the 2011 and 2012 TOMs using parallel minimum (pmin). If two nodes are highly connected in both years, the pmin TOM value for the node pair will be high. If two nodes are highly connected in only a single year, or if two nodes are unconnected in both years, the pmin TOM value will be low. The consensus TOM was then used as input for average-linkage hierarchical clustering using the hclust function and TOM dissimilarity (1-consensusTOM) to identify clusters as in Langfelder and Horvath (2016). We used *cutreeDynamic* as part of WGCNA to identify branch boundaries for consensus modules using deepSplit=2, cutHeight=0.995, and minClusterSize=10. It is during this step that oligotypes are assigned to modules if their TOM-based topology is similar in both years; those oligotypes lacking a conserved topology across both 2011 and 2012 SparCC networks are excluded from having membership to a particular module. The large gaps in the 2010 data set spanning 74 and 51 days in winter and late spring, respectively, would have been problematic for this consensus TOM step where inconsistent topology might have arisen from missing data points.

Following module detection, we calculated the module eigengene (ME) (that is, first principal component) for each module using the WGCNA script *multiSetMEs,* which performs a principal component analysis using the oligotype abundance profiles of each module. We used centered log-ratio (clr) transformed abundance data with a pseudocount of 1e-6 during this step to ensure subcompositional coherence using the R package compositions (van den Boogaart *et al.*, 2014). The calculated MEs can then serve as a representative of each module’s collective abundance profile. We then again performed average-linkage clustering of the MEs using *consensusMEDissimilarity* and hclust to identify modules sufficiently similar for merging using *mergeCloseModules* with default cutHeight=0.25. New MEs of merged modules were calculated within this merging step. An overview of our network methods can be found in Supplementary Figure S2. SparCC correlation and *P*-value matrices are available at https://github.com/mc68462/medNS/tree/v0.3/data alongside WGCNA R code at https://github.com/mc68462/medNS/blob/v0.3/wgcna.R. Network graphs were filtered using igraph and visualized with Gephi (Bastian, Heymann and Jacomy, 2009).

#### Detection of seasonally structured modules

Using each of the MEs from 2011 and 2012, we performed a redundancy analysis (RDA) to extract variation in the MEs that could be explained by the measured physicochemical variables. Because we performed RDA on the MEs and not the OTU abundance data directly, we refer to this method as an indirect gradient analysis (Legendre and Legendre, 1998; Buttigieg and Ramette, 2014a). Prior to RDA, physicochemical variables were standardized to *Z*-scores using the R package vegan (Oksanen *et al.*, 2015). Spearman correlations and *P*-values between MEs and environmental variables were performed with the base R function cor.test from the stats package (R Core Team, 2008). *P*-values were corrected for multiple testing using the base R function, p.adjust, employing the method of Benjamini and Hochberg (1995). MEs and physicochemical variables measured in this study display seasonal patterns over time. We define season boundaries according to their astronomical definition.

#### Intersection of 2011 and 2012 module networks

Consensus module detection with WGCNA identifies clusters of oligotypes that are connected and share similar neighbors across both years. To quantify the degree to which correlations between oligotypes are similar within a particular module network in 2011 versus 2012, we performed a graph intersection using the R package igraph (Csardi and Nepusz, 2006). We first extracted the SparCC networks for each consensus module and then performed a filtration step based on the strength of the SparCC correlation between oligotypes. Rather than apply a single arbitrary threshold across all modules, we performed an analysis to determine at which threshold (>abs(r)=0.5) we could trim the edges of each individual module network without losing connectedness with the main graph component (Žure *et al.*, 2017). In other words, we wanted to maximize edge filtration without breaking apart the entire module network. We tested a range of absolute value thresholds from 0 to 1 using igraph. Following the filtration of edge weights, we performed graph intersection to obtain a single network graph per consensus module containing only vertices and edges that are strongly correlated in both 2011 and 2012.

## Results

### Physicochemical and phytoplankton data

Chl *a* concentrations reveal a succession of three prominent bloom periods in 2010 with progressively weaker and delayed chl *a* peaks in 2011 and 2012 (Supplementary Figure S1). Overall diatoms tend to dominate the climax of spring and summer blooms, with the exception of marine raphidophyte *Chattonella,* which conspicuously replaces diatoms in the spring of 2012. Although the absolute abundance of these major phytoplankton groups is valuable, there are vast differences in cell sizes (see Teeling *et al.*, 2016). Abundance is thus not a reliable indicator of how much each phytoplankton group contributes to the organic substrate pool. We take chl *a* as a proxy for the eutrophic conditions characteristic of phytoplankton blooms.

### Bacterioplankton diversity

Patterns of recurrence in oligotype relative abundance were observed across the three sampled years. Supplementary Figure S3 showcases the oligotype diversity, recurrence and seasonality of major clades. *Alphaproteobacteria* (A), *Gammaproteobacteria* (B) and *Flavobacteriia* (C) comprise 73% of all oligotypes. This value might be higher were it not for the under-representation of SAR11, resulting from primer bias. All three classes increase in relative abundance each spring and summer with markedly higher oligotype diversity within *Gammaproteobacteria* and *Flavobacteriia.* Supplementary Figure S3 D-I also includes additional classes that are recurrent, but occur in relatively lower abundances. *Acidimicrobiia* (D)*, Betaproteobacteria* (E) and *Verrucomicrobia* (H) are present throughout much of the year. *Euryarchaeota* (F) and *Planctomycetes* (I) appear later in the year, whereas *Epsilonproteobacteria* (G) appear very briefly each year during bloom seasons. Overall, oligotype abundance patterns of all major clades exhibit striking patterns of recurrent oligotype diversity, with a notable delay in the timing of the bacterioplankton response to spring phytoplankton blooms in 2011 and 2012 compared with 2010 within bloom responders *Alphaproteobacteria*, *Gammaproteobacteria* and *Flavobacteriia*.

**Figure 1:**
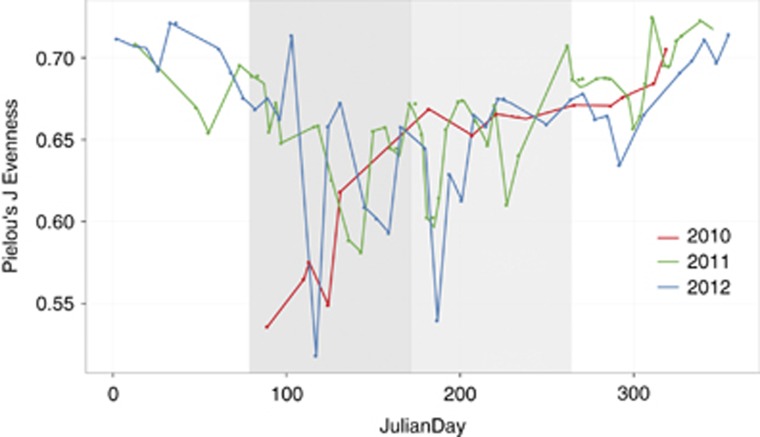
Spring and summer harbor communities of skewed evenness. Pielou’s J metric of evenness is represented on the *y* axis and Julian days on the *x* axis. A value of 1.0 represents total community evenness. Spring and summer are denoted by the dark and light gray areas, respectively. Line colors denote year: red=2010, blue=2011, green=2012.

We interpolated and extrapolated alpha diversity using the R package iNEXT (Chao *et al.*, 2014; Hsieh *et al.*, 2016). Asymptotic diversity estimates alongside related statistics are included in Supplementary Table S5. Rarefaction curves calculated from proportional oligotype richness data (*q*=0) fell between 1249 and 2879 and did not reach saturation (Supplementary Figure S4A). However, exponential Shannon (expShannon) and inverse Simpson (invSimpson) diversity estimates revealed much reduced *effective* diversity. ExpShannon estimates reported 47–307 effective species and invSimpson estimates reported 6–96 effective species throughout the year (Supplementary Figure S4B). Each spring and summer, invSimpson estimates fall as low as 6 and 14 total species, respectively. Pielou’s J evenness displays this skew in alpha diversity each spring and summer, followed by a return to winter baseline levels of evenness (Figure 1). Overall our analyses indicate that the vast majority of the annual bacterioplankton diversity at Helgoland can be explained by fewer than 100 effective oligotypes, as per the maximum effective diversity calculated with invSimpson. The top 100 most abundant oligotypes comprise 64% of the total 16S rRNA gene amplicon abundance. However, the under-representation of SAR11 in our datasets might have deflated measures of alpha diversity compared with previous work employing the same principle of oligotyping at a coastal sampling site (Eren *et al.*, 2013; Fuhrman *et al.*, 2017). Supplementary Figure S5 displays the relative abundances of the three SAR11 oligotypes we identified (reaching a minimum abundance of 1%) detected with our primers. Our previous study employing SAR11-specific fluorescence *in situ* hybridization probes (FISH) revealed that SAR11 peaked at ~50% of community abundance shortly prior to spring bloom events; our 16S rRNA gene abundances during this same time period top off at ~8% relative community abundance. Although SAR11 abundance is significantly under-represented in our data set, the pattern over time recapitulates FISH counts from the spring study of Teeling *et al.*, 2016.

### Patterns of oligotype prevalence

**Figure 2:**
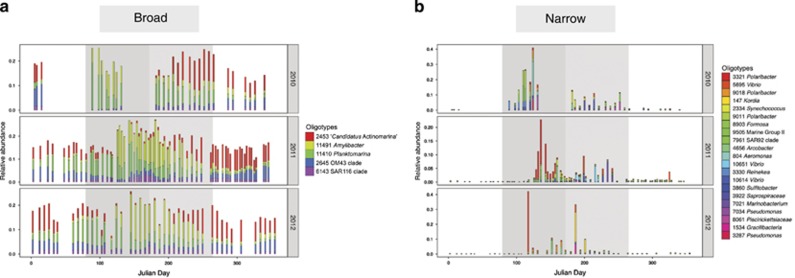
Oligotypes show distinct patterns of prevalence each year. Relative abundance plots show 'broad' (**a**) and 'narrow' (**b**) categories of oligotype prevalence patterns. Julian days are represented on the *x* axis and oligotype relative abundance is represented as a fraction on the *y* axis. Spring and summer are denoted by the dark and light gray areas, respectively. Broad oligotypes are defined by >75% prevalence and narrow oligotypes are defined by <10% prevalence. All five broad oligotypes identified in Figure S6 are shown in panel A and collectively occupy ~20% of the total community throughout the year. Narrow oligotypes reaching an abundance of 5% in at least one sample are shown in panel B and are enriched in spring and summer. Julian days with no bar indicate the absence of a sample point. Table S6 contains a full list of identified broad and narrow oligotypes.

Examining the prevalence of a given oligotype revealed two groups marked by broad and narrow distributions over time (Supplementary Figure S6,Supplementary Table S6). A clear partition is seen for five abundant and broadly prevalent oligotypes present in >75% of samples and include: actinobacterial ‘*Candidatus* Actinomarina’ oligotype_2453, alphaproteobacterial *Amylibacter* oligotype_11491, *Planktomarina* oligotype_11410 and the SAR116 clade oligotype_6143, and the betaproteobacterial OM43 clade oligotype_2645. These five oligotypes occupy ~20% of the total community relative abundance throughout the year (Figure 2a). *Planktomarina* oligotype_11410 is 100% identical to the formally described and highly abundant *Planktomarina temperata* RCA23 strain isolated from the southern North Sea (Giebel *et al.*, 2011, 2013), and is known to occupy a large proportion of temperate and polar water masses (Selje *et al.*, 2004). In total we identified 298 oligotypes enriched during spring and summer, which display a narrow prevalence comprising a diverse collection of primarily *Gammaproteobacteria* and *Flavobacteriia* (Figure 2b). Narrowly prevalent *Gammaproteobacteria* include *Vibrio* oligotypes 5695, 10651 and 10614, SAR92 clade oligotype_7961, *Reinekea* oligotype_3330 and *Aeromonas* oligotype_804. Narrowly prevalent *Flavobacteriia* include *Polaribacter* oligotypes 3321 and 9018, *Kordia* oligotype_147 and *Formosa* oligotype_8903.

### Oligotype switching

**Figure 3:**
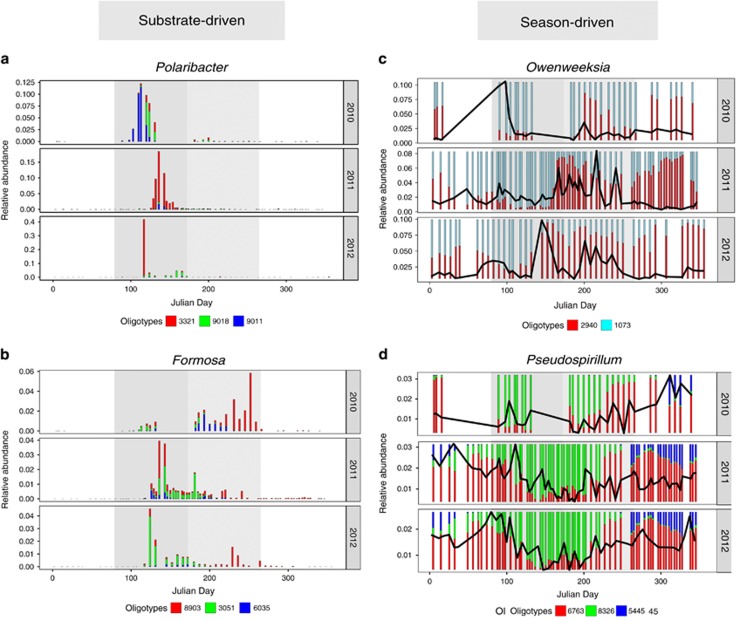
Season and phytoplankton substrate-driven oligotype switching is observed between oligotypes with >97% identity. Relative abundance bar plots show examples of switches in oligotype dominance. Julian days are represented on the *x* axis and total oligotype relative abundance is represented as a fraction on the *y* axis. Spring and summer are denoted by the dark and light gray areas, respectively. *Flavobacteriia* genera *Polaribacter* (**a**) and *Formosa* (**b**) exhibit potential phytoplankton substrate-driven changes in dominance over short time intervals between oligotypes with 99.5% identity. Genera *Owenweeksia*, (**c**, *Flavobacteriia*) and *Pseudospirillum* (**d**, *Gammaproteobacteria*) exhibit sinusoidal seasonal changes in dominance between oligotypes with >97% identity, visible when relative abundance is scaled to 1 (stacked bars). The total cumulative relative abundance of displayed oligotypes in **c** and **d** is represented by the black line (*y* axis).

Algal-derived organic biomass dominates spring and summer substrate sources as proxied by chl *a*. During spring and summer, oligotypes with 99.5% identity within the flavobacterial genera *Polaribacter* (Figure 3a) and *Formosa* (Figure 3b) exhibited rapid shifts in dominance, potentially driven by phytoplankton-derived substrate supply. Genera *Owenweeksia* VIS6 (*Flavobacteriia*) and *Pseudospirillum* (*Gammaproteobacteria*) exhibited seasonally driven changes in oligotype dominance with a more gradual sinusoidal abundance pattern. In late spring/early summer, the dominant *Owenweeksia* oligotype_1073 switched to oligotype_2940 (Figure 3c), with which it is 98.4% identical, and three oligotypes classified as *Pseudospirillum* share 97.4% identity but exhibit recurrent seasonal patterns (Figure 3d). These examples display the ability of MED to resolve subtle and ecologically meaningful seasonal shifts in diversity.

### Identification of recurrent and seasonally structured module networks

The goal of our network analysis was to identify whether oligotypes could be found within recurrent sub-networks. The TOM-based method we used in WGCNA consensus module detection takes into account correlations and topology to identify clusters of oligotypes that are connected and shared similar neighbors in both 2011 and 2012 SparCC networks. Oligotypes lacking conserved topology would correspondingly lack module membership, however, all 3664 network oligotypes were found within consensus modules. During module detection, we chose to merge similar modules based on the dissimilarity of their MEs (see Materials and Methods). Prior to merging, we identified 11 consensus modules and after merging we obtained a total of seven modules (Supplementary Figure S7). Despite our setting allowing a minimum module size of 10, all detected merged modules contained a large number of oligotypes with between 254 and 628 members (Table 1); pre-merged modules contained between 76 and 622 member oligotypes (data not shown). The vast majority of SparCC correlations between oligotypes within each module were positive; negative correlations tended to be weak (abs(r)<0.5) (Supplementary Figure S8). Given the low values of inverse Simpson diversity in spring and summer (6 and 14, respectively), the use of a compositionality-robust network construction method was particularly important to avoid spurious negative correlations during these periods of low diversity (Weiss *et al.*, 2016).

**Figure 4:**
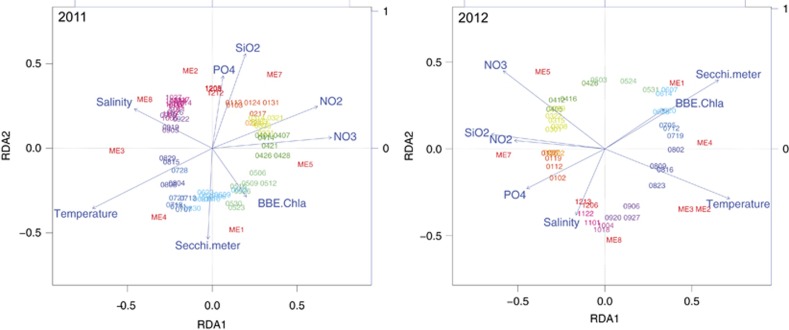
Module eigengenes (MEs) are structured by seasonally fluctuating environmental variables. Redundancy analysis (RDA) of MEs against measurements of physicochemical variables revealed a repeating cyclical seasonal pattern of MEs and sample time points across 2011 (left panel) and 2012 (right panel). Sample time points are denoted by date (mmdd). MEs are differentially driven by oligotrophic conditions (high inorganic nutrient concentrations: PO_4_^3−^, SiO_2_, NO_2_^−^ and NO_3_^−^), eutrophic conditions (high chl *a* and Secchi disk depth), salinity and/or temperature. Sample points are colored by month: red shades denote winter, green denotes spring, blue denotes summer and purple denotes autumn.

**Figure 5:**
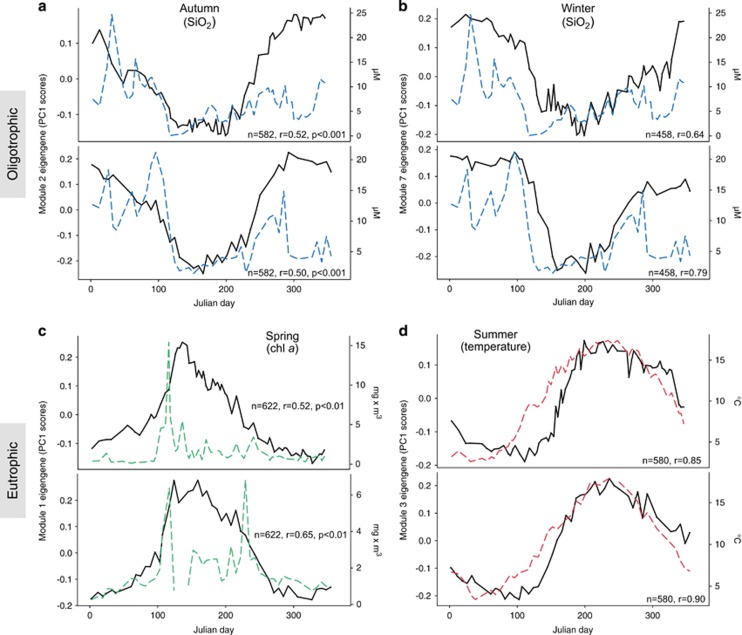
Module eigengenes (MEs) display recurrent correlations with environmental variables across 2011 and 2012. MEs (black lines) serve as a proxy for the collective abundance of its member oligotypes and are overlaid with their most strongly correlated environmental variable (dashed lines) (**a**–**d**) across Julian days represented on the *x* axis. Inorganic nutrients are denoted by blue dashed lines, chlorophyll *a* by green dashed lines and temperature by red dashed lines. ME trends are represented on the left *y* axis and environmental variables on the right *y* axis. *n* and *r* correspond to the total number of module oligotypes and Spearman correlation, respectively. *P*-values (p) are adjusted employing the method of Benjamini and Hochberg (1995).

**Figure 6:**
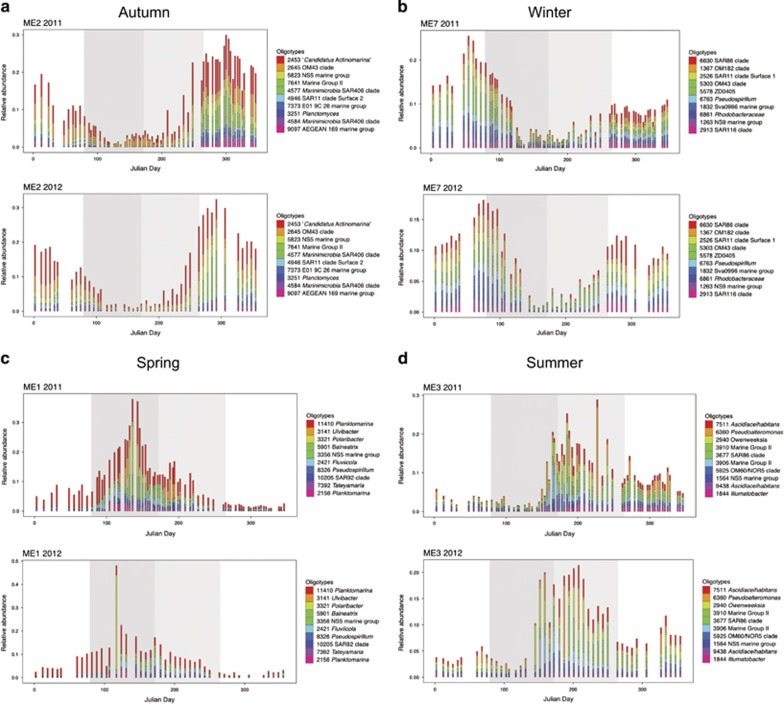
Module oligotype profiles reveal shifts in diversity patterns depending on the presence of bloom conditions. Relative abundance plots (**a**–**d**) show four of the seven identified consensus modules and display the top 10 most abundant module oligotypes. Modules are described according to their season of highest abundance: ME2=‘Autumn’ (**a**), ME7=‘Winter’ (**b**), ME1=‘Spring’ (**c**) and ME3='Summer' (**d**). Julian days are represented on the *x* axis and relative abundance is represented as a fraction on the *y* axis. Spring and summer are denoted by the dark and light gray areas, respectively.

The construction of modules allowed us to identify oligotypes that co-vary. Using MEs of each module, we performed an RDA against physicochemical variables. RDAs revealed a repeating cyclical seasonal pattern of MEs and sample time points across 2011 and 2012 (Figure 4). Modules are differentially driven by oligotrophic and eutrophic conditions (Supplementary Table S7). Positive correlations with inorganic nutrients (PO_4_^3−^, SiO_2_, NO_2_^−^ and NO_3_^−^) indicate that heterotrophs are likely subsisting on low concentrations of low molecular weight organic compounds during periods of low primary productivity, whereas positive correlations to chl *a* indicate a community of heterotrophs that can reach high abundance during periods rich in high molecular weight phytoplankton-derived organic compounds. In Figure 5 we display the MEs of module 2, 7, 1 and 3 against the environmental variable with which they are most strongly and/or significantly correlated. MEs serve as a proxy for the collective abundance of its member oligotypes and for most modules, their pattern closely tracks with seasonal fluctuations of environmental variables. Module diversity (Figure 6) mirrors the seasonal shifts we identified with measures of alpha diversity (Figure 1 and Supplementary Figure S4). We describe each module based on the season in which member oligotypes appear in the highest abundance. Modules of autumn (module 2) and winter (module 7) tend to harbor more diverse and even communities driven by oligotrophic conditions, whereas modules of spring (module 1) and summer (module 3) tend to be driven by eutrophic conditions and harbor fewer oligotypes that reach high relative abundance (Figure 6).

Here we highlight module dynamics characteristic of winter, spring, summer and autumn; Supplementary Figures S9 and S10 contain corresponding plots for all modules. Winter module 7 correlates to oligotrophic conditions marked by high concentrations of inorganic nutrients like SiO_2_ (Figure 5b) and is represented by equal relative abundances of gammaproteobacterial *Oceanospirillales* SAR86 clade oligotype_6630 and OM182 clade oligotype_1367, alphaproteobacterial SAR11 clade oligotype_2526 and betaproteobacterial OM43 clade oligotype_5303 (Figure 6b). Spring module 1 is driven by increases in chl *a* concentrations as active bloom conditions commence (Figure 5c) and is dominated by alphaproteobacterial *Planktomarina* oligotype_11410, flavobacterial *Ulvibacter* oligotype_3141 and *Polaribacter* oligotype_3321, and gammaproteobacterial *Balneatrix* oligotype_5901 (Figure 6c). The diversity in this spring module is highly skewed by the high relative abundance of *Planktomarina* and particularly by *Polaribacter,* which has previously been shown to display copiotrophic growth on algal substrates (Cottrell and Kirchman, 2016). *Polaribacter* is narrowly prevalent at abundances of 15–40% of the total community (Figure 2b and 3a). Summer module 3 displays a relationship with temperature (Figure 5d), and we begin to observe an increase in community diversity and evenness (Figure 6d). Alphaproteobacterial *Ascidiaceihabitans* oligotype_7511, flavobacterial *Owenweeksia* oliogotype_2940 (Figure 3c) and euryarchaeotal Marine Group II (*Euryarchaeota*) oligotypes 3910 and 3906 comprise the more abundant community members. Although community evenness has partially rebounded since spring, it is within this module that we find the gammaproteobacterial *Pseudoalteromonas* oligotype_6360 repeatedly reaching 10–20% of the relative community abundance from an abundance near zero (Figure 6d). Prior to module merging (see Materials and Methods), this *Pseudoalteromonas* oligotype was clustered into module 11 alongside other *Gammaproteobacteria* (for example, *Vibrio* and *Arcobacter*), which display a similar feast-and-famine abundance pattern (Supplementary Figure S11). Autumn module 2 marks a return to oligotrophic conditions characterized by correlations with inorganic nutrients (Figure 5a). Autumn module 2 is dominated by actinobacterial ‘*Candidatus* Actinomarina’ oligotype_2453 (Figure 6a). Community evenness is slightly skewed by this abundant oligotype, but overall, the community begins a reset to winter baseline conditions of high evenness driven by oligotrophic conditions.

### Conservation of module network topology

To evaluate the conservation of module network topology across 2011 and 2012, we extracted the unfiltered SparCC networks for each module and filtered out weak correlations. We employed different correlation thresholds for each module in order to preserve module network integrity (Table 1). The visualization of network graph deconstruction across different correlation thresholds reveals information about how increasing filtration stringency affects graph structures (Supplementary Figure S12). Most modules exhibit a clear threshold where the networks break up into multiple smaller components, as evidenced by the sharp peak in the number of individual graph components appearing between SparCC correlation values of 0.5 and 0.75. *P*-values for filtered graphs were all ⩽0.001 with the exception of a handful at ⩽0.01. Individual filtered modules from 2011 and 2012 are shown in Supplementary Figure S13. Network graphs make clear the seasonal dynamics of module diversity with even community abundances during autumn and winter and highly skewed abundances spanning spring and summer. Module networks are conserved, even during highly dynamic periods in spring and summer; the same abundant oligotypes are found across both years with similar patterns of topology.

**Figure 7:**
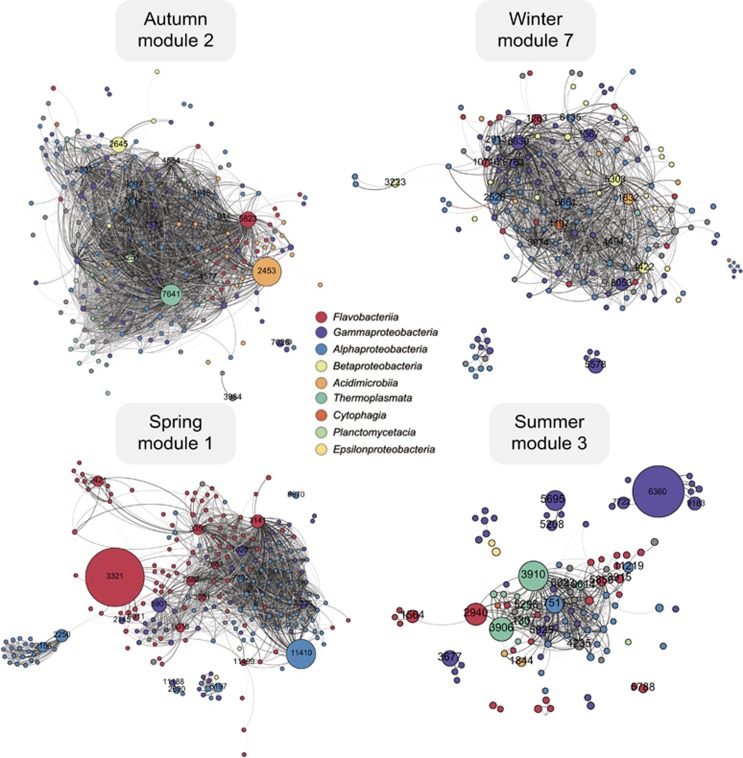
The intersection of module networks from 2011 and 2012. Individual module networks from 2011 and 2012 were filtered to exclude edges with weak SparCC correlations (Table 1), followed by the removal of vertices which became disconnected from all other vertices (degree=0). Corresponding filtered module networks were then intersected using igraph (Csardi and Nepusz, 2006) to obtain a single graph representative of oligotypes that are strongly correlated across 2011 and 2012 module networks. The percentage of shared vertices and edges is shown in Table 1. Vertex size is represented as the average maximum observed oligotype abundance in 2011 and 2012 and is labeled with an oligotype id if that abundance is >1%. Vertices are colored by class-level taxonomy. Oligotype taxonomic identities can be found in Supplementary Table S4. Edges are colored from light gray to black to denote weak to strong SparCC correlations, respectively. Decreases in community evenness can be seen in bloom compared with non-bloom seasons.

In order to quantify the degree of similarity between 2011 and 2012 filtered module network correlations, we performed a graph intersection, which is the equivalent of retaining the overlapping regions of a Venn diagram. Intersected module networks are depicted in Figure 7 and Supplementary Figure S14, and represent oligotypes and correlations that are conserved in both years. Between 53 and 75% of vertices and between 32 and 49% of edges were shared across 2011 and 2012 (Table 1). Overall, this indicates that module correlation structures are similar across years, sharing on average 70% of vertices and 43% of edges. However, summer module 3 has the lowest overlap in shared network edges and vertices (Supplementary Figure S15), and contains disconnected graph components largely comprised of *Gammaproteobacteria* oligotypes (Figure 7). Network topology suggests that correlation patterns within the summer module are less conserved across years.

## Discussion

### Resiliency and microdiversity of microbial communities in the North Sea

We examined the dynamics of free-living bacterioplankton communities at Helgoland Roads over three consecutive years using highly resolved 16S rRNA gene oligotypes. Throughout the course of our study, annual patterns of microdiversity within the free-living bacterioplankton community appeared largely conserved from year to year (for example, Supplementary Figure S3). We used network module eigengenes (MEs) to represent potential guilds of co-varying bacterioplankton, the members of which may exploit similar resources, may interact or may tolerate similar environmental conditions. MEs showed recurrent correlations with seasonally fluctuating environmental parameters across 2011 and 2012 that were broadly defined by oligotrophic and eutrophic conditions (Figure 5). However, as previously found in marine environments (Eren *et al.*, 2013; Ward *et al.*, 2017), we identified seasonal patterns of microdiversity. For example, *Cryomorphaceae Owenweeksia*-classified oligotypes 1073 and 2940 share 98.4% sequence identity but display seasonality in oligotype dominance (Figure 3c), and they are found within two separate module networks (Figure 6).

Higher frequency shifts in oligotype dominance over short time intervals within Flavobacteriia *Polaribacter* and *Formosa* (Figures 3a and b), are more likely owing to subtle shifts in algal-derived substrate that cannot be resolved by measures of chl *a*. There is evidence that as in the human gut (Koropatkin *et al.*, 2012), polysaccharides can shape marine microbial niches upon their release from algal cells (Xing *et al.*, 2015). Even subtle differences in metabolic pathways and enzyme deployment strategies can differentiate niches of organisms that consume the same algal-derived substrate (Hehemann *et al.*, 2016). Endeavors to evaluate the response of bacterioplankton to specific types of dissolved organic matter molecules suggests that an improved resolution in the characterization of organic substrate is required to uncover the drivers of these more fine-scale shifts in diversity during blooms (Lucas *et al.*, 2016).

In an analysis of prevalence, we identified a distinct grouping of broadly and narrowly abundant oligotypes, indicating the ability of some taxa to exploit resources available throughout much of the year versus those specialized on more ephemeral resource patches. For example, broadly prevalent oligotype_11410 (Figure 2a) is 100% identical to the *Planktomarina temperata* RCA23 strain (Giebel *et al.*, 2013) known to widely occupy temperate and polar water masses (Selje *et al.*, 2004). Although strains with identical 16S rRNA genes can harbor widely different gene content, oligotype_11410 may correspond to *P. temperata* RCA23, which was originally isolated near our sampling site in the southern North Sea (Giebel *et al.*, 2011). Genomic analyses suggest that *P. temperata* RCA23 is a versatile strain with a high proportion of ATP-binding cassette family transporters (Voget *et al.*, 2015) and is capable of aerobic anoxygenic photosynthesis (Giebel *et al.*, 2013). The RCA23 strain thus appears well adapted to nutrient-poor conditions with a relatively streamlined genome compared with other members of the *Roseobacter* clade, but has also demonstrated high *in situ* activity across nearly its entire genome during high-nutrient spring bloom conditions in the North Sea (Voget *et al.*, 2015). If this strain does in fact correspond to oligotype_11410, its genomic capabilities would explain the broad prevalence patterns we observe during oligotrophic and eutrophic conditions, even as more copiotrophic *Flavobacteriia* like the narrowly prevalent *Polaribacter* oligotypes rise in abundance. Here the partition of niche space likely derives from differential preferences for high molecular weight and low molecular weight compounds; alphaproteobacterial *P. temperata* might target low molecular weight molecules via ATP-binding cassette transporters, whereas flavobacterial *Polaribacter* might deploy specialized carbohydrate-active enzymes and transport strategies for the rapid uptake of high molecular weight algal polysaccharides (Xing *et al.*, 2015; Reintjes *et al.*, 2017).

The predictable response of the network modules to environmental variables reveals that each year, resilient autumn and winter bacterioplankton communities re-emerge following dynamic spring and summer bloom disturbance events. Moreover, despite differences in the composition of blooming phytoplankton in 2011 and 2012, there appears in both years a similarly structured secondary bloom within the free-living bacterioplankton community with a marked drop in the conservation of summer module topology across years.

### Ecological strategies during spring and summer

Given the patterns of recurrence we have identified across 2011 and 2012, we present a hypothesis regarding the differences in diversity, dynamics and network topology we observed in spring versus summer communities. During winter at Helgoland, system-level productivity is low (for example, low chl *a* (Supplementary Figure S1) and total cell counts (Supplementary Figure S16)). As viruses and grazers require a critical encounter frequency with their prey to proliferate (Wilcox and Fuhrman, 1994; Cram *et al.*, 2016), Teeling *et al.*, (2016) proposed that spring Helgoland bacterioplankton communities might be subject to less predation following grazer suppression in winter. The KtW (kill-the-winner hypothesis) predicts a balance between bottom-up competition for growth during periods of low productivity and defense against top–down predators during periods of high productivity (Thingstad and Lignell, 1997; Thingstad, 2000; Våge *et al.*, 2013; Thingstad *et al.*, 2014). We hypothesize that spring bacteria responding to the first releases of algal-derived organic matter are competitive for the fresh substrates and capable of rapid growth before grazers and viruses targeting their populations take hold. In contrast, each summer is marked by high productivity (maximal chl *a* and total cell counts) and thus high potential grazing pressure (KtW). Furthermore, there is evidence from aquatic systems that an increase in predator richness has a positive effect on bacterial richness and evenness (Saleem *et al.*, 2012) stemming primarily from the widespread distribution of resources and resulting niche complementarity (Tilman *et al.*, 1997; Loreau, 1998; Cardinale *et al.*, 2002). The result is an increase in ecosystem biomass and richness (Mulder *et al.*, 2001). We thus hypothesize that the increase in bacterioplankton community diversity and evenness in summer might arise from an increase in grazing diversity and pressure. Any discussion of diversity and evenness must, however, consider the inevitable PCR-bias of the primer set used for sequence retrieval, in our case suppressing SAR11.

*Flavobacteriia* and *Gammaproteobacteria* are both specialized in the degradation of algal-derived high molecular weight organic matter, are both enriched during diatom-dominated blooms and both display copiotrophic capabilities (Pernthaler and Amann, 2005; Cottrell and Kirchman, 2016). However, their abundance patterns differ between spring and summer. *Flavobacteriia* and *Gammaproteobacteria* in spring of 2011 and 2012 tend to maintain their abundance for several weeks and do not reappear in summer (Figure 6c). Conversely, summer copiotrophic *Gammaproteobacteria* appear to be governed by a different set of underlying dynamics that result in a pronounced feast-and-famine lifestyle. *Gammaproteobacteria* have been shown to be opportunistic in nature, harboring the ability to rapidly respond to algal-derived substrates from low abundance (Pernthaler and Amann, 2005). They are also known to be subject to higher viral pressure than their aerobic anoxygenic phototrophic counterparts (for example, *Bacteroidetes* and alphaproteobacterial *Roseobacter* and SAR11) (Ferrera *et al.*, 2011). In our data set, *Pseudoaltermonas* oligotype_6360 reaches high abundance in summer module 3 in both years (Figure 7) and exhibits repeated cycles of rapid growth and decline, much like *Psychrobacter* oligotype_5208 and *Vibrio* oligotype_5696 (Supplementary Figure S11), which is also known to be susceptible to lytic phage infection (for example, Kellogg *et al.*, 1995). Our network analysis revealed that these *Gammaproteobacteria* oligotypes tend to cluster together in a loose connection to the main network graph (Figure 7, Supplementary Figure S13). Although many marine clades are subject to host-phage dynamics that shape diversity abundance over time (Needham *et al.*, 2017), we hypothesize that frequency-dependent phage infections (KtW) (Thingstad and Lignell, 1997; Thingstad, 2000) might contribute to the irregular abundance patterns we have identified in summer *Gammaproteobacteria*. Such dynamics might explain the destabilization of network conservation across the 2 years investigated here. These shifts in abundance patterns from spring to summer might stem in part from a shift in temperature optima of distinct bacterial taxa as suggested previously (Lucas *et al.*, 2015), but we hypothesize that it might also arise from the shift in the ecological strategies required for proliferation under an increasingly complex and dense web of biochemical activity and top–down pressure.

## Conclusion and outlook

We conclude that cycles of deterministic selection pressure driven by stable seasonal forcing has, through the induction of recurrent patters in resource availability, predator–prey dynamics and microbial interactions, allowed for the assemblage of largely stable and resilient free-living microbial communities throughout both non-bloom and bloom conditions. Although we expected the community to return to its stable winter state following bloom events, we did not expect communities with such similar network topologies to emerge during dynamic bloom events. However, we acknowledge that the two years used in network analysis might have been two considerably similar years with regards to the timing of the onset of primary and secondary blooms, which were delayed in 2011 and 2012 compared with 2010. Additional years of data are required in order to capture and predict the more complete range of possible bloom scenarios and community successions that respond to differences in the extent of grazer die-off in winter, bloom timing and phytoplankton composition. Furthermore, the diversity, dynamics and resilience of the particle-attached bacterioplankton fraction are likely very different from that of the free-living fraction measured here. Molecular characterization of particle-attached bacteria and eukaryotic phytoplankton is required to evaluate resilience and identify potential interactions across size fractions and between trophic levels. Permanent, automated systems for global ocean sampling offer a solution for this type of data collection (for example, Davies and Field, 2012; Soltwedel *et al.*, 2013; Davies *et al.*, 2014), which is essential to effectively monitor, describe, model and manage ocean ecosystem diversity. In combination with methods like MED to resolve patterns of microdiversity based on the 16S rRNA gene, these long-term ecological monitoring sites provide opportunities to detect previously undescribed archaeal and bacterial diversity (Buttigieg and Ramette, 2014b).

Nevertheless, in this study we have highlighted the value of multi-year studies in identifying recurrent modular community associations in a dynamic coastal environment. Our use of a sub-compositionally coherent network construction method affords a higher level of confidence in the correlations we measured here between oligotypes, particularly during the low diversity periods of spring and summer, setting forth a foundation for the exploration of ecologically meaningful associations between community members. The modules we present thus serve as targets for further inquiry including genomic characterization and laboratory-based experiments in order to elucidate the mechanisms and potential microbial interactions underlying recurrent patterns of microdiversity.

## Figures and Tables

**Table 1:** Module network graph statistics

*Module*	*Season*	*Total oligotypes*	*SparCC cor threshold 2011*^a^	*SparCC cor threshold 2012*^a^	*Intersecting vertices (%)*^b^	*Intersecting edges (%)*^b^
2	Autumn	584	0.51	0.58	72.6	44.7
7	Winter	458	0.53	0.55	74.9	48.7
5	Winter–Spring	628	0.51	0.56	74.6	35.9
1	Spring	622	0.61	0.61	72.7	40.2
4	Spring–Summer	538	0.62	0.57	74.1	48.7
3	Summer	580	0.52	0.59	53	32
8	Summer–Autumn	254	0.5	0.55	67	48.2

a SparCC correlation thresholds were determined according to an analysis of network deconstruction across different thresholds (Supplementary Figure S12).

b Intersecting vertices and edges were calculated using igraph (Csardi and Nepusz, 2006).

## References

[bib1] Aitchison J. (2003) The statistical analysis of compositional data. Blackburn Press: Caldwell, NJ, USA.

[bib2] Apprill A, McNally S, Parsons R, Weber L. (2015). Minor revision to V4 region SSU rRNA 806 R gene primer greatly increases detection of SAR11 bacterioplankton. Aquat Microb Ecol 75: 29–137.

[bib3] Azam F, Ammerman JW. (1984) Flows of energy and materials in marine ecosystems. In: Fasham MJR (ed). Springer: Boston, MA, USA.

[bib4] Azam F, Fenchel T, Field J, Gray J, Meyer-Reil L, Thingstad F. (1983). The ecological role of water-column microbes in the sea. Mar Ecol Prog Ser 10: 257–263.

[bib5] Barberán A, Bates ST, Casamayor EO, Fierer N. (2012). Using network analysis to explore co-occurrence patterns in soil microbial communities. ISME J 6: 343–351. 21900968 10.1038/ismej.2011.119 PMC3260507

[bib6] Bastian M, Heymann S, Jacomy M. (2009), An open source software for exploring and manipulating networks.

[bib7] Benjamini Y, Hochberg Y. (1995). Controlling the false discovery rate: a practical and powerful approach to multiple testing. J R Stat Soc Series B 57: 289–300.

[bib8] Berry D, Widder S. (2014). Deciphering microbial interactions and detecting keystone species with co-occurrence networks. Front Microbiol 5: 219. 24904535 10.3389/fmicb.2014.00219 PMC4033041

[bib9] van den Boogaart KG, Tolosana R, Bren M. (2014), compositions: Compositional data analysis.

[bib10] Pawlowsky-Glahn V, Buccianti A. (ed. (2011) Compositional data analysis: theory and applications. Wiley: Chichester, West Sussex, UK, pp 400.

[bib11] Buchan A, LeCleir GR, Gulvik CA, González JM. (2014). Master recyclers: features and functions of bacteria associated with phytoplankton blooms. Nat Rev Microbiol 12: 686–698. 25134618 10.1038/nrmicro3326

[bib12] Buttigieg PL, Ramette A. (2014a). A guide to statistical analysis in microbial ecology: a community-focused, living review of multivariate data analyses. FEMS Microbiol Ecol 90: 543–550. 25314312 10.1111/1574-6941.12437

[bib13] Buttigieg PL, Ramette A. (2014b). Biogeographic patterns of bacterial microdiversity in Arctic deep-sea sediments (HAUSGARTEN, Fram Strait). Front Microbiol 5: 660. 25601856 10.3389/fmicb.2014.00660 PMC4283448

[bib14] Cardinale BJ, Palmer MA, Collins SL. (2002). Species diversity enhances ecosystem functioning through interspecific facilitation. Nature 415: 426–429. 11807553 10.1038/415426a

[bib15] Chao A, Gotelli NJ, Hsieh TC, Sander EL, Ma KH, Colwell RK et al. (2014). Rarefaction and extrapolation with Hill numbers: a framework for sampling and estimation in species diversity studies. Ecol Monogr 84: 45–67.

[bib16] Chow C-ET, Sachdeva R, Cram JA, Steele JA, Needham DM, Patel A et al. (2013). Temporal variability and coherence of euphotic zone bacterial communities over a decade in the Southern California Bight. ISME J 7: 2259–2273. 23864126 10.1038/ismej.2013.122 PMC3834854

[bib17] Cottrell M, Kirchman DL. (2016). Transcriptional control in marine copiotrophic and oligotrophic bacteria with streamlined genomes. Appl Environ Microbiol 82: 6010–6018. 27474718 10.1128/AEM.01299-16 PMC5038029

[bib18] Cram JA, Parada AE, Fuhrman JA. (2016). Dilution reveals how viral lysis and grazing shape microbial communities. Limnol Oceanogr 61: 889–905.

[bib19] Csardi G, Nepusz T. (2006). The igraph software package for complex network research. InterJournal, Complex Systems, pp 1695.

[bib20] Cuskin F, Lowe EC, Temple MJ, Zhu Y, Cameron EA, Pudlo NA et al. (2015). Human gut *Bacteroidetes* can utilize yeast mannan through a selfish mechanism. Nature 517: 165–169. 25567280 10.1038/nature13995 PMC4978465

[bib21] Davies N, Field D. (2012). Sequencing data: a genomic network to monitor Earth. Nature 481: 45. 10.1038/481145a 22237100

[bib22] Davies N, Field D, Amaral-Zettler L, Clark M, Deck J, Drummond A et al. (2014). The founding charter of the genomic observatories network. GigaScience 3: 2. 24606731 10.1186/2047-217X-3-2 PMC3995929

[bib23] Ducklow HW, Carlson CA. (1992). Oceanic bacterial production. Adv Microb Ecol 12: 113–181.

[bib24] Eren AM, Maignien L, Sul WJ, Murphy LG, Grim SL, Morrison HG et al. (2013). Oligotyping: differentiating between closely related microbial taxa using 16S rRNA gene data. Methods Ecol Evol 4: 1111–1119. 10.1111/2041-210X.12114 PMC3864673 24358444

[bib25] Eren AM, Vineis JH, Morrison HG, Sogin ML. (2013). A filtering method to generate high quality short reads using Illumina paired-end technology. PLoS One 8: e66643. 10.1371/journal.pone.0066643 PMC3684618 23799126

[bib26] Eren AM, Morrison HG, Lescault PJ, Reveillaud J, Vineis JH, Sogin ML. (2015). Minimum entropy decomposition: unsupervised oligotyping for sensitive partitioning of high-throughput marker gene sequences. ISME J 9: 968–979. 25325381 10.1038/ismej.2014.195 PMC4817710

[bib27] Ferrera I, Gasol JM, Sebastián M, Hojerová E, Koblížek M. (2011). Comparison of growth rates of aerobic anoxygenic phototrophic bacteria and other bacterioplankton groups in coastal Mediterranean waters. Appl Environ Microbiol 77: 7451–7458. 21724878 10.1128/AEM.00208-11 PMC3209150

[bib28] Friedman J, Alm EJ. (2012). Inferring correlation networks from genomic survey data. PLoS Comp Biol 8: e1002687. 10.1371/journal.pcbi.1002687 PMC3447976 23028285

[bib29] Fuhrman JA, Cram JA, Needham DM. (2015). Marine microbial community dynamics and their ecological interpretation. Nat Rev Microbiol 13: 133–146. 25659323 10.1038/nrmicro3417

[bib30] Fuhrman JA, Hewson I, Schwalbach MS, Steele JA, Brown MV, Naeem S. (2006). Annually reoccurring bacterial communities are predictable from ocean conditions. PNAS 103: 13104–13109. 16938845 10.1073/pnas.0602399103 PMC1559760

[bib31] Giebel H-A, Kalhoefer D, Gahl-Janssen R, Choo Y-J, Lee K, Cho J-C et al. (2013). *Planktomarina temperata* gen. nov, sp. nov, belonging to the globally distributed RCA cluster of the marine Roseobacter clade, isolated from the German Wadden Sea. Int J Syst Evol Microbiol 63: 4207–4217. 23793856 10.1099/ijs.0.053249-0

[bib32] Giebel H-A, Kalhoefer D, Lemke A, Thole S, Gahl-Janssen R, Simon M et al. (2011). Distribution of Roseobacter RCA and SAR11 lineages in the North Sea and characteristics of an abundant RCA isolate. ISME J 5: 8–19. 20596072 10.1038/ismej.2010.87 PMC3105675

[bib33] Gilbert JA, Field D, Swift P, Newbold L, Oliver A, Smyth T et al. (2009). The seasonal structure of microbial communities in the Western English Channel. Environ Microbiol 11: 3132–3139. 19659500 10.1111/j.1462-2920.2009.02017.x

[bib34] Gilbert JA, Steele JA, Caporaso JG, Steinbrück L, Reeder J, Temperton B et al. (2012). Defining seasonal marine microbial community dynamics. ISME J 6: 298–308. 21850055 10.1038/ismej.2011.107 PMC3260500

[bib35] Hehemann J-H, Arevalo P, Datta MS, Yu X, Corzett CH, Henschel A et al. (2016). Adaptive radiation by waves of gene transfer leads to fine-scale resource partitioning in marine microbes. Nat Commun 7: 12860. 27653556 10.1038/ncomms12860 PMC5036157

[bib36] Hsieh TC, Ma KH, Chao A. (2016). iNEXT: an R package for rarefaction and extrapolation of species diversity (Hill numbers). Methods Ecol Evol 7: 1451–1456.

[bib37] Kearse M, Moir R, Wilson A, Stones-Havas S, Cheung M, Sturrock S et al. (2012). Geneious Basic: an integrated and extendable desktop software platform for the organization and analysis of sequence data. Bioinformatics 28: 1647–1649. 22543367 10.1093/bioinformatics/bts199 PMC3371832

[bib38] Kellogg CA, Rose JB, Jiang SC, Thurmond JM, Paul JH. (1995). Genetic diversity of related vibriophages isolated from marine environments around Florida and Hawaii, USA. Mar Ecol Prog Ser 120: 89–98.

[bib39] Koropatkin NM, Cameron EA, Martens EC. (2012). How glycan metabolism shapes the human gut microbiota. Nat Rev Microbiol 10: 323–335. 22491358 10.1038/nrmicro2746 PMC4005082

[bib40] Langfelder P, Horvath S. (2016). Tutorials for the WGCNA package for R: II Consensus network analysis of liver expression data, female and male mice.

[bib41] Langfelder P, Horvath S. (2008). WGCNA: an R package for weighted correlation network analysis. BMC Bioinformatics 9: 559. 19114008 10.1186/1471-2105-9-559 PMC2631488

[bib42] Legendre L, Le Fèvre J. (1989) Hydrodynamic singularities as controls of recycled versus export production in oceans. In: Berger WH, Smetacek VS, Wefer G. (ed). Productivity of the ocean: present and past. Wlley: Chichester, UK, pp 65–83.

[bib43] Legendre P, Legendre L. (1998) Numerical Ecology. 2nd edn. Elsevier: Amsterdam, The Netherlands.

[bib44] Loreau M. (1998). Biodiversity and ecosystem functioning: a mechanistic model. PNAS 95: 5632–5636. 9576935 10.1073/pnas.95.10.5632 PMC20430

[bib45] Lu H-P, Yeh Y-C, Sastri AR, Shiah F-K, Gong G-C, Hsieh C. (2016). Evaluating community-environment relationships along fine to broad taxonomic resolutions reveals evolutionary forces underlying community assembly. ISME J 10: 2867–2878. 27177191 10.1038/ismej.2016.78 PMC5148199

[bib46] Lucas J, Koester I, Wichels A, Niggemann J, Dittmar T, Callies U et al. (2016). Short-term dynamics of North Sea bacterioplankton-dissolved organic matter coherence on molecular level. Front Microbiol 7: 321. 27014241 10.3389/fmicb.2016.00321 PMC4791370

[bib47] Lucas J, Wichels A, Teeling H, Chafee M, Scharfe M, Gerdts G. (2015). Annual dynamics of North Sea bacterioplankton: seasonal variability superimposes short-term variation. FEMS Microbiol Ecol 91: fiv099. 26298013 10.1093/femsec/fiv099

[bib48] Martin M. (2011). Cutadapt removes adapter sequences from high-throughput sequencing reads. EMBnet.journal 17: 10–12.

[bib49] Martiny JBH, Jones SE, Lennon JT, Martiny AC. (2015). Microbiomes in light of traits: a phylogenetic perspective. Science 350: aac9323. 26542581 10.1126/science.aac9323

[bib50] McMurdie PJ, Holmes S. (2013). phyloseq: an R package for reproducible interactive analysis and graphics of microbiome census data. PLoS One 8: e61217. 23630581 10.1371/journal.pone.0061217 PMC3632530

[bib51] Mulder CPH, Uliassi DD, Doak DF. (2001). Physical stress and diversity-productivity relationships: the role of positive interactions. PNAS 98: 6704–6708. 11371612 10.1073/pnas.111055298 PMC34416

[bib52] Needham DM, Fuhrman JA. (2016). Pronounced daily succession of phytoplankton, archaea and bacteria following a spring bloom. Nat Microbiol 29: 16005. 10.1038/nmicrobiol.2016.5 27572439

[bib53] Needham DM, Sachdeva R, Fuhrman JA. (2017). Ecological dynamics and co-occurrence among marine phytoplankton, bacteria and myoviruses shows microdiversity matters. ISME J 11: 1614–1629. 28398348 10.1038/ismej.2017.29 PMC5520149

[bib54] Oksanen J, Blanchet FG, Kindt R, Legendre P, Minchin PR, O’Hara RB et al. (2015), vegan: Community ecology package. R package version 2.3-0.

[bib55] Parada AE, Needham DM, Fuhrman JA. (2016). Every base matters: assessing small subunit rRNA primers for marine microbiomes with mock communities, time series and global field samples. Environ Microbiol 18: 1403–1414. 26271760 10.1111/1462-2920.13023

[bib56] Pernthaler J, Amann R. (2005). Fate of heterotrophic microbes in pelagic habitats: focus on populations. Microbiol Mol Biol Rev 69: 440–461. 16148306 10.1128/MMBR.69.3.440-461.2005 PMC1197807

[bib57] Philippot L, Andersson SGE, Battin TJ, Prosser JI, Schimel JP, Whitman WB et al. (2010). The ecological coherence of high bacterial taxonomic ranks. Nat Rev Microbiol 8: 523–529. 20531276 10.1038/nrmicro2367

[bib58] Raabe T, Wiltshire KH. (2009). Quality control and analyses of the long-term nutrient data from Helgoland Roads, North Sea. J Sea Res 61: 3–16.

[bib59] R Core Team.. (2008) R: A language and environment for statistical computing. R Foundation for Statistical Computing: Vienna, Austria.

[bib60] Reintjes G, Arnosti C, Fuchs BM, Amann R. (2017). An alternative polysaccharide uptake mechanism of marine bacteria. ISME J 11: 1640–1650. 28323277 10.1038/ismej.2017.26 PMC5520146

[bib61] Saleem M, Fetzer I, Dormann CF, Harms H, Chatzinotas A. (2012). Predator richness increases the effect of prey diversity on prey yield. Nat Commun 3: 1305. 23250435 10.1038/ncomms2287

[bib62] Selje N, Simon M, Brinkhoff T. (2004). A newly discovered Roseobacter cluster in temperate and polar oceans. Nature 427: 445–448. 14749832 10.1038/nature02272

[bib63] Soltwedel T, Schauer U, Boebel O, Nothig E-M, Bracher A, Metfies K et al. (2013). FRAM - FRontiers in Arctic marine Monitoring: Visions for permanent observations in a gateway to the Arctic Ocean. In MTS/IEEE OCEANS - Bergen. IEEE, pp 1–7.

[bib64] Steinberg DK, Carlson CA, Bates NR, Johnson RH, Michaels AF, Knap AH. (2001). Overview of the US JGOFS Bermuda Atlantic Time-series Study (BATS): a decade-scale look at ocean biology and biochemistry. Deep Sea Res II 48: 1405–1447.

[bib65] Teeling H, Fuchs BM, Becher D, Klockow C, Gardebrecht A, Bennke CM et al. (2012). Substrate-controlled succession of marine bacterioplankton populations induced by a phytoplankton bloom. Science 336: 608–611. 22556258 10.1126/science.1218344

[bib66] Teeling H, Fuchs BM, Bennke CM, Krüger K, Chafee M, Kappelmann L et al. (2016). Recurring patterns in bacterioplankton dynamics during coastal spring algae blooms. eLife 5: e11888. 27054497 10.7554/eLife.11888 PMC4829426

[bib67] Thingstad TF. (2000). Elements of a theory for the mechanisms controlling abundance, diversity, and biogeochemical role of lytic bacterial viruses in aquatic systems. Limnol Oceanogr 45: 1320–1328.

[bib68] Thingstad TF, Våge S, Storesund JE, Sandaa R-A, Giske J. (2014). A theoretical analysis of how strain-specific viruses can control microbial species diversity. PNAS 111: 7813–7818. 24825894 10.1073/pnas.1400909111 PMC4040589

[bib69] Thingstad T, Lignell R. (1997). Theoretical models for the control of bacterial growth rate, abundance, diversity and carbon demand. Aquat Microb Ecol 13: 19–27.

[bib70] Thomas F, Hehemann J-H, Rebuffet E, Czjzek M, Michel G. (2011). Environmental and gut *Bacteroidetes*: The food connection. Front Microbiol 2: 93. 21747801 10.3389/fmicb.2011.00093 PMC3129010

[bib71] Tilman D, Lehman CL, Thompson KT. (1997). Plant diversity and ecosystem productivity: theoretical considerations. PNAS 94: 1857–1861. 11038606 10.1073/pnas.94.5.1857 PMC20007

[bib72] Utter DR, Mark Welch JL, Borisy GG. (2016). Individuality, stability, and variability of the plaque microbiome. Front Microbiol 7: 564. 27148241 10.3389/fmicb.2016.00564 PMC4840391

[bib73] Våge S, Storesund JE, Thingstad TF. (2013). Adding a cost of resistance description extends the ability of virus-host model to explain observed patterns in structure and function of pelagic microbial communities. Environ Microbiol 15: 1842–1852. 23331773 10.1111/1462-2920.12077

[bib74] Voget S, Wemheuer B, Brinkhoff T, Vollmers J, Dietrich S, Giebel H-A et al. (2015). Adaptation of an abundant Roseobacter RCA organism to pelagic systems revealed by genomic and transcriptomic analyses. ISME J 9: 371–384. 25083934 10.1038/ismej.2014.134 PMC4303631

[bib75] Ward CS, Yung C-M, Davis KM, Blinebry SK, Williams TC, Johnson ZI et al. (2017). Annual community patterns are driven by seasonal switching between closely related marine bacteria. ISME J 11: 1412–1422. 28234350 10.1038/ismej.2017.4 PMC5437356

[bib76] Weiss S, Van Treuren W, Lozupone C, Faust K, Friedman J, Deng Y et al. (2016). Correlation detection strategies in microbial data sets vary widely in sensitivity and precision. ISME J 10: 1669–1681. 26905627 10.1038/ismej.2015.235 PMC4918442

[bib77] Wickham H. (2009) ggplot2: elegant graphics for data analysis. Springer: New York, NY, USA.

[bib78] Wilcox RM, Fuhrman JA. (1994). Bacterial viruses in coastal seawater: lytic rather than lysogenic production. Mar Ecol Prog Ser 114: 35–45.

[bib79] Wiltshire KH, Kraberg A, Bartsch I, Boersma M, Franke H-D, Freund J et al. (2009). Helgoland Roads, North Sea: 45 years of change. Estuaries Coasts 33: 295–310.

[bib80] Xing P, Hahnke RL, Unfried F, Markert S, Huang S, Barbeyron T et al. (2015). Niches of two polysaccharide-degrading *Polaribacter* isolates from the North Sea during a spring diatom bloom. ISME J 9: 1410–1422. 25478683 10.1038/ismej.2014.225 PMC4438327

[bib81] Žure M, Fernandez-Guerra A, Munn CB, Harder J. (2017). Geographic distribution at subspecies resolution level: closely related *Rhodopirellula* species in European coastal sediments. ISME J 11: 478–489. 27801907 10.1038/ismej.2016.123 PMC5270564

