# Supplementary material for: Recurrent patterns of microdiversity in a temperate coastal marine environment
Source: ISME J. 2017 Oct 24;12(1):237–52. doi: 10.1038/ismej.2017.165 (PMC5739018; doi:10.1038/ismej.2017.165)
Supplement: Supplementary Figure S1 [file ismej2017165x8.pdf]

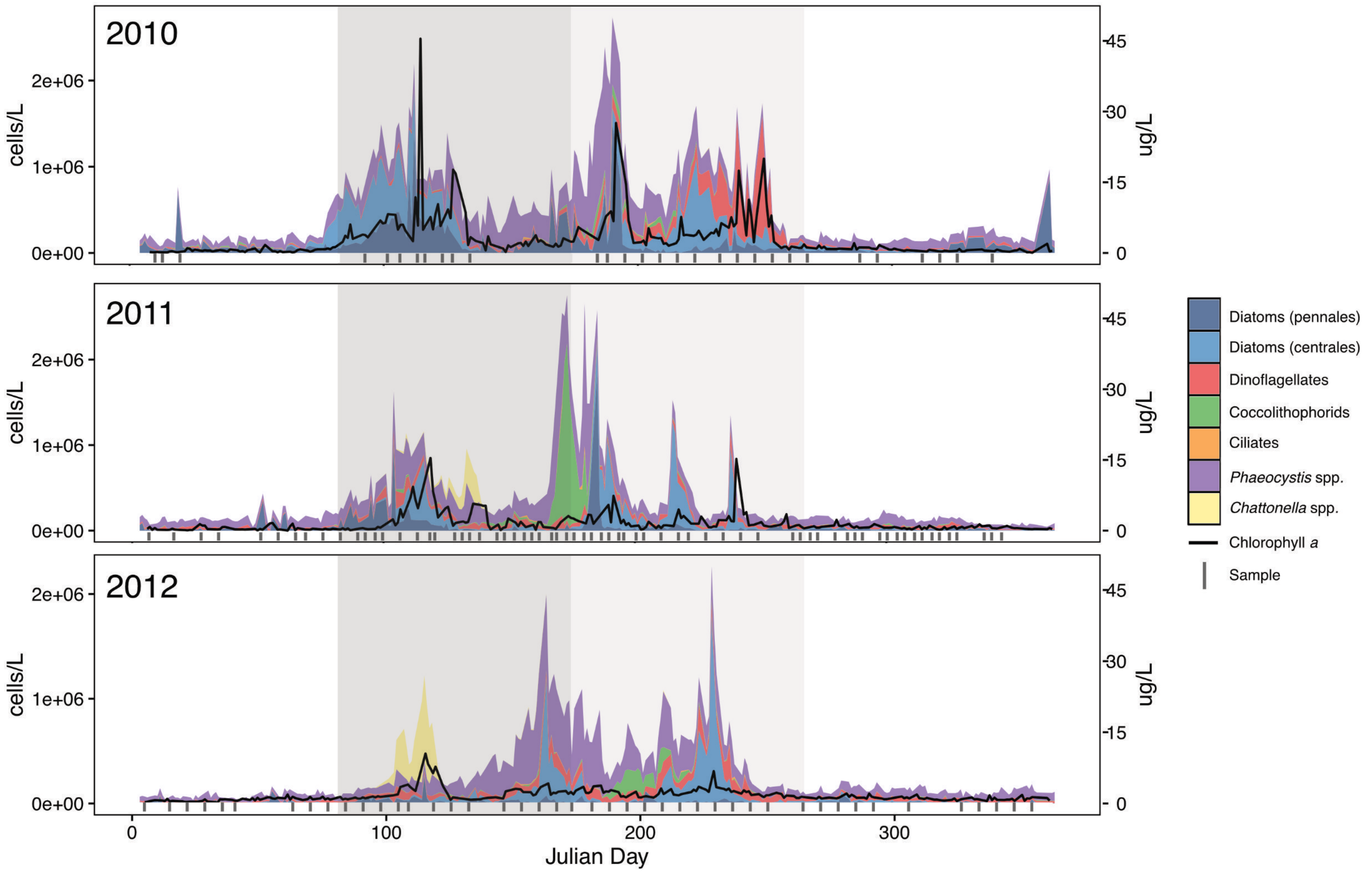

Figure S1. Phytoplankton bloom intensity and composition varies year to year. Stacked area colors represent cell counts of dominant phytoplankton groups (cells/L) on the left y-axis across Julian days on the x-axis: dark blue = Diatoms (pennales), light blue = Diatoms (centrales), red = Dinoflagellates, green = Coccolithophorids, orange = Ciliates, purple = *Phaeocystis* spp. (prymnesiophyte), yellow = *Chattonella* spp. (raphidophyte). Spring and summer are denoted by the dark and light grey areas, respectively. Black lines denote chlorophyll a concentration (µg/L) on the right y-axis. Vertical grey lines below the area plot denote sampling time points.
