# Supplementary material for: Recurrent patterns of microdiversity in a temperate coastal marine environment
Source: ISME J. 2017 Oct 24;12(1):237–52. doi: 10.1038/ismej.2017.165 (PMC5739018; doi:10.1038/ismej.2017.165)
Supplement: Supplementary Figure S2 [file ismej2017165x9.pdf]

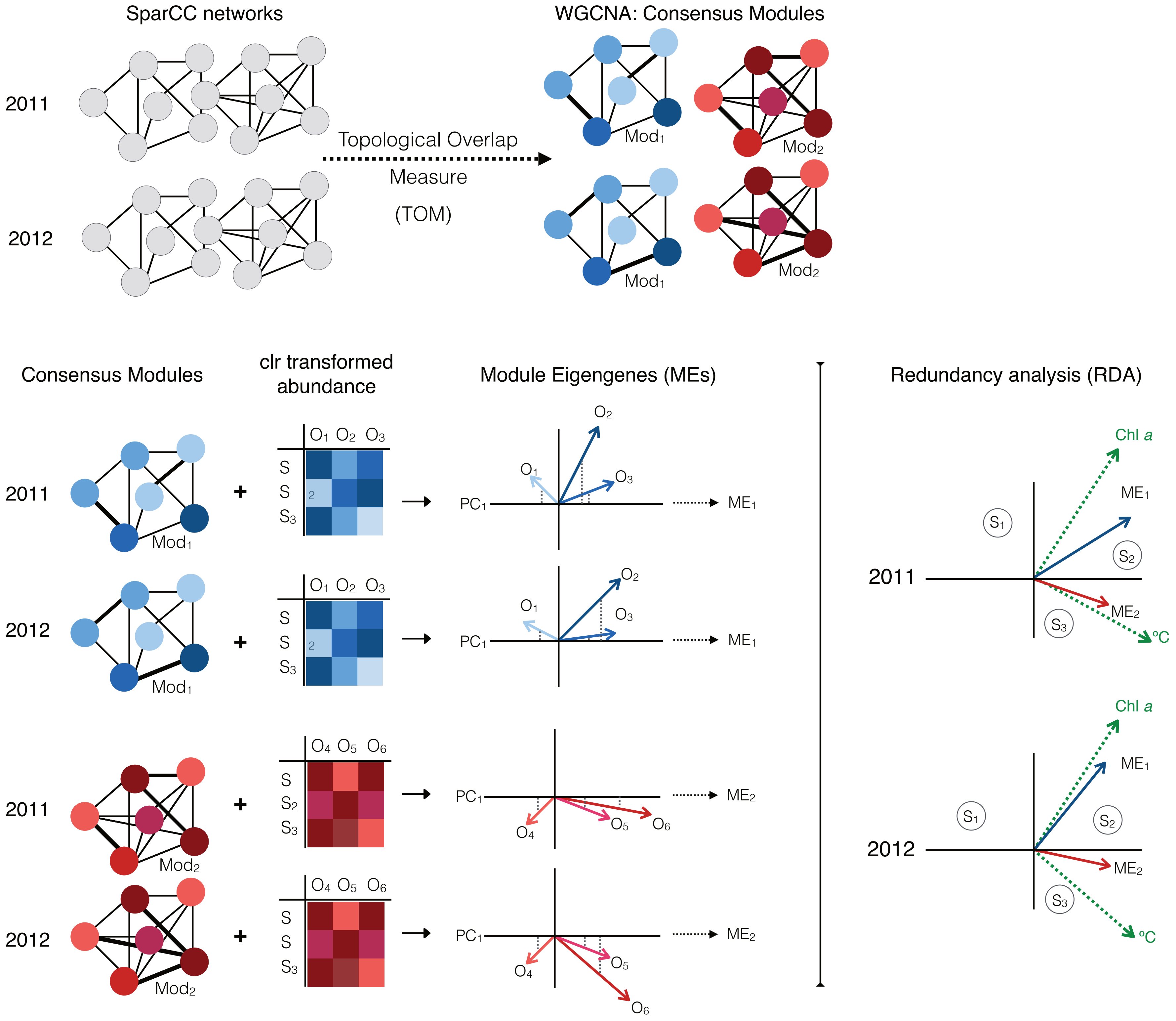

Figure S2. Network analysis methods. Independent networks for 2011 and 2012 16S rRNA gene datasets were constructed using SparCC (Kurtz et al. 2015). We then employed WGCNA (Langfelder et al. 2008) to identify consensus modules that were present in both 2011 and 2012 using a topological overlap matrix (TOM). The membership of oligotypes is thus the same for each consensus module identified in both years (represented by network vertices) but those member oligotypes may exhibit different SparCC correlations (represented by network edge weights). We calculated module eigengenes (MEs) using clr-transformed oligotype abundance data in a principle component analysis for each consensus module. MEs are represented by the first principle component of each module. We then used each ME in a redundancy analysis to determine which measured environmental variables explain the most variation within each ME.
