# Supplementary material for: Recurrent patterns of microdiversity in a temperate coastal marine environment
Source: ISME J. 2017 Oct 24;12(1):237–52. doi: 10.1038/ismej.2017.165 (PMC5739018; doi:10.1038/ismej.2017.165)
Supplement: Supplementary Figure S3 [file ismej2017165x10.pdf]

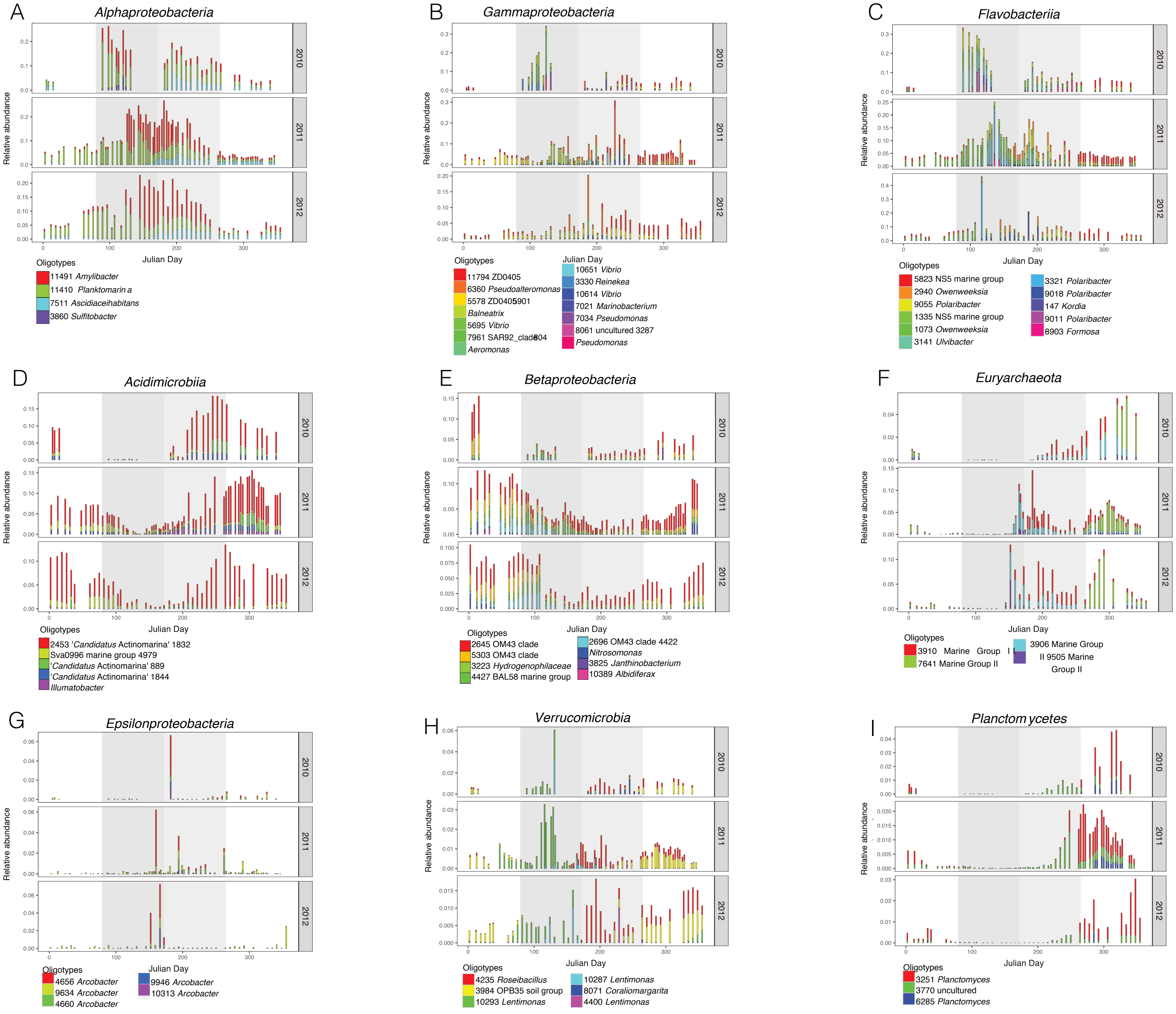

Figure S3. Oligotype diversity and abundance of major bacterioplankton classes reveals widespread recurrence. *Alphaproteobactera* (A), *Gammaproteobacteria* (B), *Flavobacteriia* (C), *Acidimicrobiia* (D), *Betaproteobacteria* (E), *Euryarchaetoa* (F), *Epsilonproteobacteria* (G), *Verrucomicrobia* (H) and *Planctomycetes* (I). Julian days are shown on the x-axis and oligotype relative abundance as a fraction on the y-axis. Spring and summer are denoted by the dark and light grey areas, respectively. Oligotypes in A, B, C and F arefiltered according to a minimum 5% abundance in at least one sample and oligotypes in D, E, G, H, and I are filtered according to aminimum 1% abundance in at least one sample.
