# Supplementary material for: Recurrent patterns of microdiversity in a temperate coastal marine environment
Source: ISME J. 2017 Oct 24;12(1):237–52. doi: 10.1038/ismej.2017.165 (PMC5739018; doi:10.1038/ismej.2017.165)
Supplement: Supplementary Figure S4 [file ismej2017165x11.pdf]

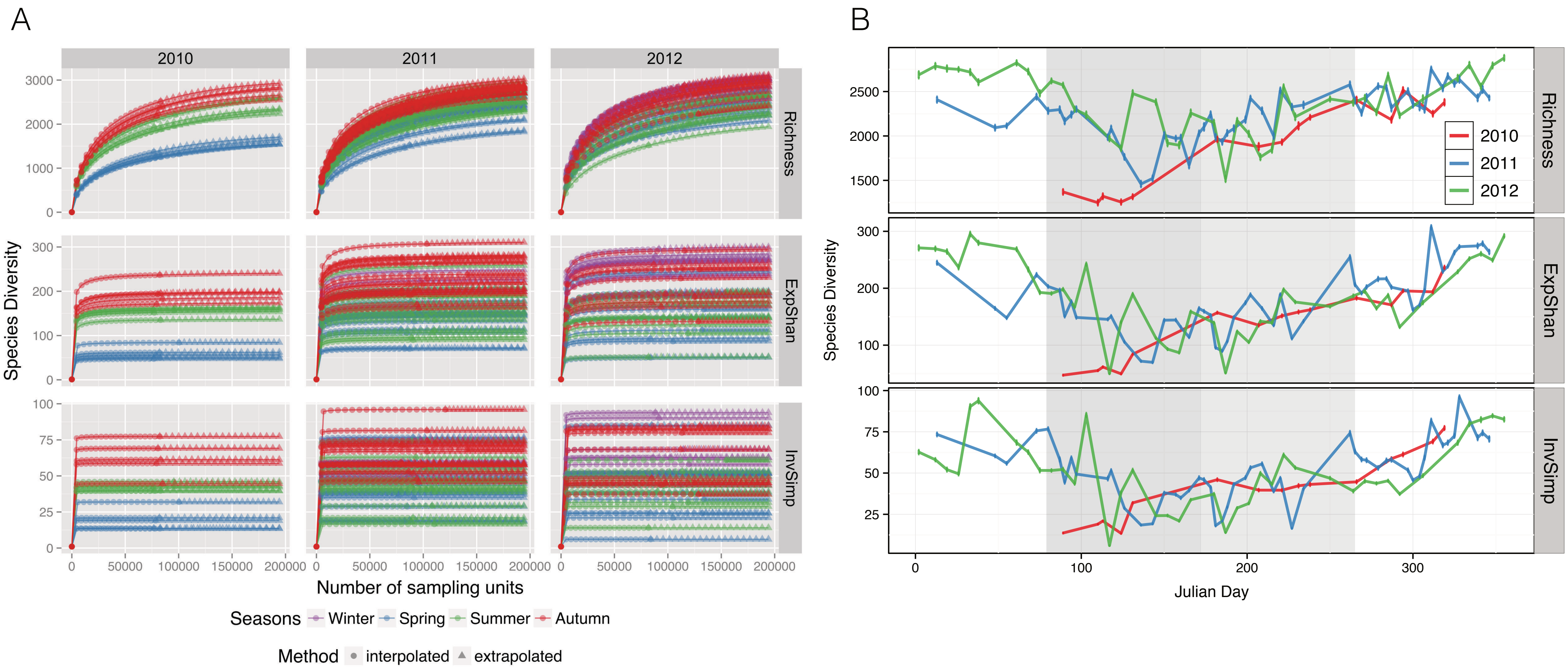

Figure S4. North Sea surface waters support streamlined archaeal and bacterial diversity. Alpha diversity is calculated using richness (q = 0), exponential Shannon (q = 1) and inverse Simpson (q = 2) diversity metrics using the R package iNEXT (Chao et al, 2014). For rarefaction curves (A), subsampling units are shown on the x-axis and the number of effective species on the y-axis. Line colors denote season: purple = winter, blue = spring, green = summer, red = autumn. Data point shapes denote method: circle = interpolated, triangle = extrapolated. Temporal changes in diversity (B) are shown with the number of effective species on the y-axis over Julian days on the x-axis. Spring and summer are denoted by the dark and light grey areas, respectively. Line colors denote year: red = 2010, blue = 2011 and green = 2012. Error bars represent upper and lower 95% confidence intervals of 50 bootstrap replicates.
