# Supplementary material for: Recurrent patterns of microdiversity in a temperate coastal marine environment
Source: ISME J. 2017 Oct 24;12(1):237–52. doi: 10.1038/ismej.2017.165 (PMC5739018; doi:10.1038/ismej.2017.165)
Supplement: Supplementary Figure S5 [file ismej2017165x12.pdf]

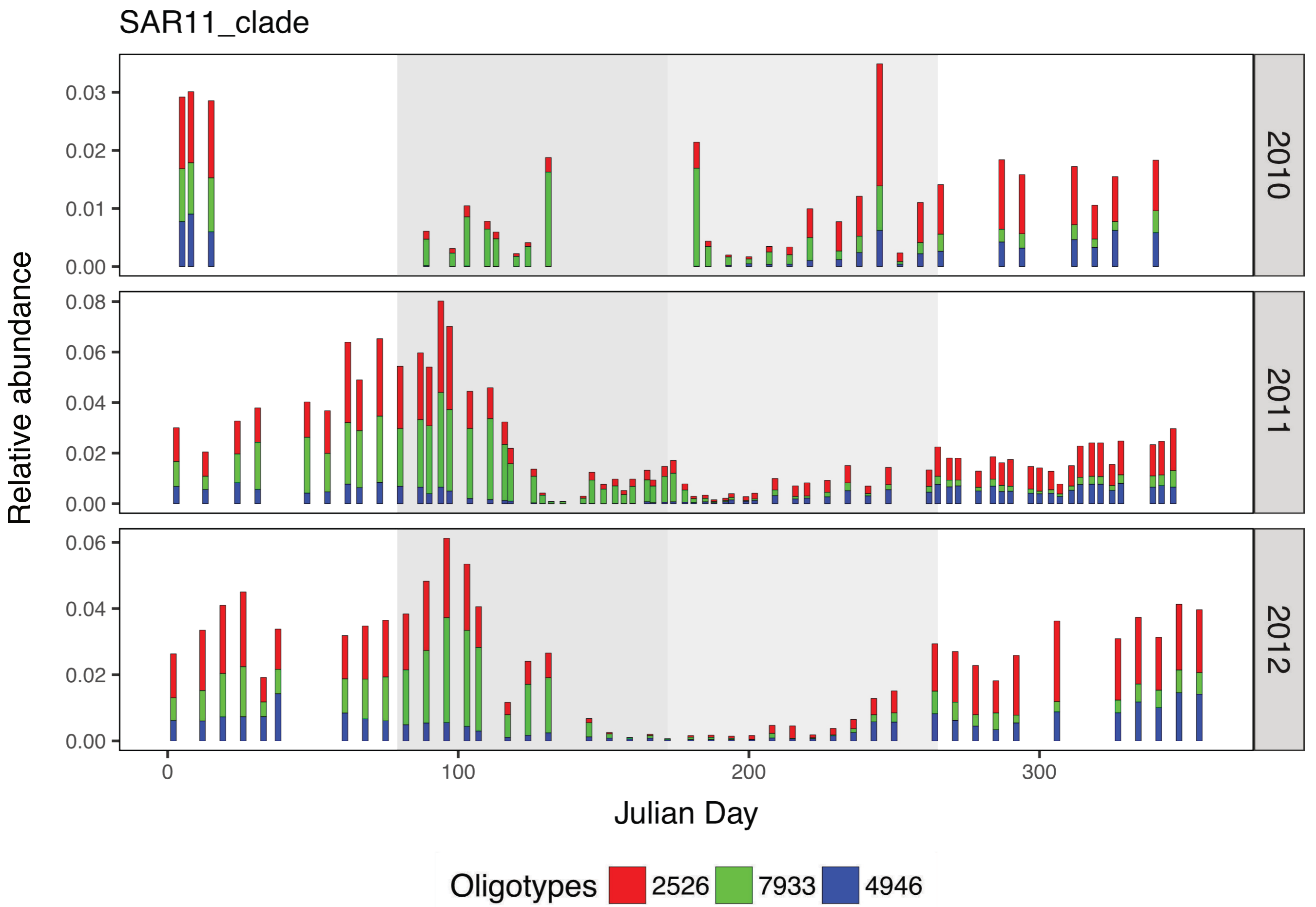

Figure S5. The alphaproteobacterial SAR11 clade is under-represented due to primer bias in the V4 16S rRNA gene primers used in this study (Parada et al. 2015). Julian days are shown on the x-axis and oligotype relative abundance as a fraction on the y-axis. Spring and summer are denoted by the dark and light grey areas, respectively. Oligotypes shown are filtered according to a minimum 1% abundance in at least one sample. Fluorescent *in situ* hybridization with SAR11-specific probes confirms this primer bias in our previous study which included the same time points from Julian day 60-160 (Teeling et al. 2016). However, relative abundance patterns presented here recapitulate the same relative patterns in Teeling et al. 2016 where SAR11 clade abundances drop sharply between Julian days 125 and 150 in 2011 and 2012.
