# Supplementary material for: Recurrent patterns of microdiversity in a temperate coastal marine environment
Source: ISME J. 2017 Oct 24;12(1):237–52. doi: 10.1038/ismej.2017.165 (PMC5739018; doi:10.1038/ismej.2017.165)
Supplement: Supplementary Figure S6 [file ismej2017165x13.pdf]

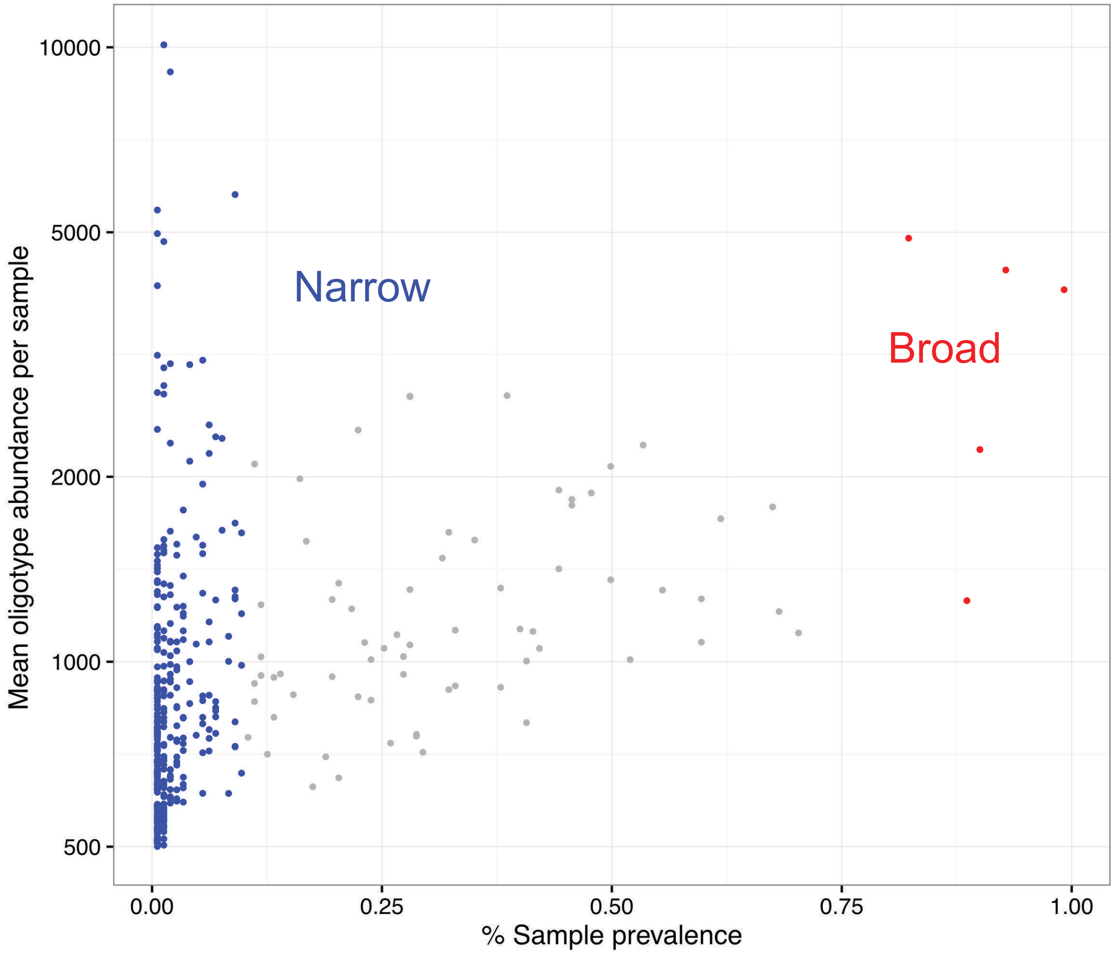

Figure S6. Oligotype prevalence. Percent oligotype prevalence (out of 142 samples across 2010-2012) is shown on the x-axis and mean oligotype abundance per sample is shown on the y-axis. Each point represents an oligotype that appears at least 500 times in one sample. The y-axis represents the average oligotype abundance per sample calculated from those samples in which that oligotype appears more than 500 times. The five ‘broad’ oligotypes (red) are defined as having ≥75% prevalence and the 298 ‘narrow’ oligotypes (blue) are defined as having ≤10% prevalence. The 68 oligotypes lacking a membership to one of these two categories are colored grey. Oligotype and taxonomic identify of broad and narrow oligotypes can be found in Table S6.
