# Supplementary material for: Recurrent patterns of microdiversity in a temperate coastal marine environment
Source: ISME J. 2017 Oct 24;12(1):237–52. doi: 10.1038/ismej.2017.165 (PMC5739018; doi:10.1038/ismej.2017.165)
Supplement: Supplementary Figure S7 [file ismej2017165x14.pdf]

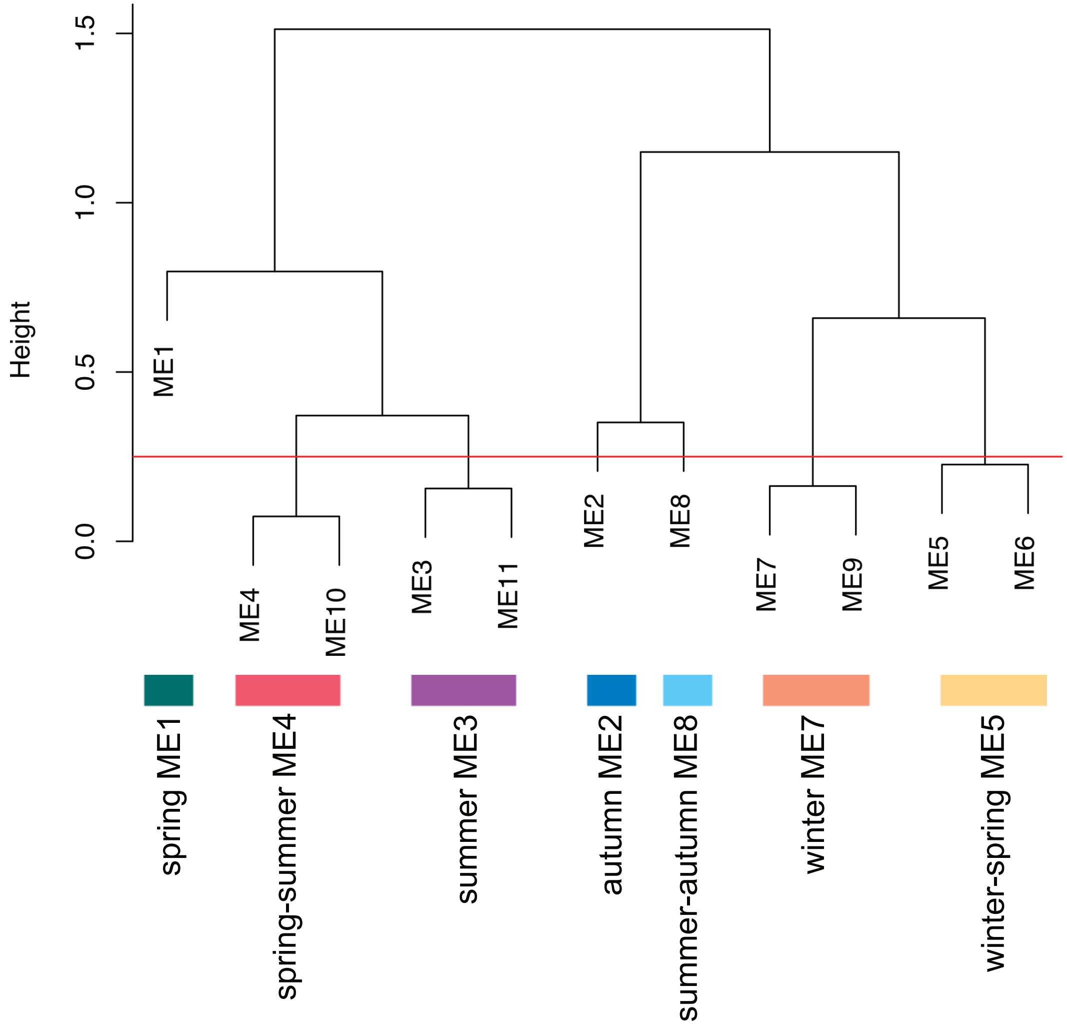

Figure S7. Clustering of consensus module eigengenes (MEs) prior to merging. Consensus MEs were clustered using average linkage clustering of ME dissimilarity with WGCNA script *consensusMEDissimilarity* (Langfeder et al. 2008). Similar modules were merged based on a default cut height of 0.25 (red line) using the *mergeCloseModules* script. ME branches below the red line were merged: ME4 and ME10, ME3 and ME11, ME7 and ME9, and ME5 and ME6 were merged into ME4, ME3, ME7 and ME5, respectively (7 total merged consensus modules). Clustering patterns reveal spring module 1to be the most distinct. Unmerged ME11 was identified as a 'feast-and-famine' module with oligotype abundance shown in Figure S11.
