# Supplementary material for: Recurrent patterns of microdiversity in a temperate coastal marine environment
Source: ISME J. 2017 Oct 24;12(1):237–52. doi: 10.1038/ismej.2017.165 (PMC5739018; doi:10.1038/ismej.2017.165)
Supplement: Supplementary Figure S8 [file ismej2017165x15.pdf]

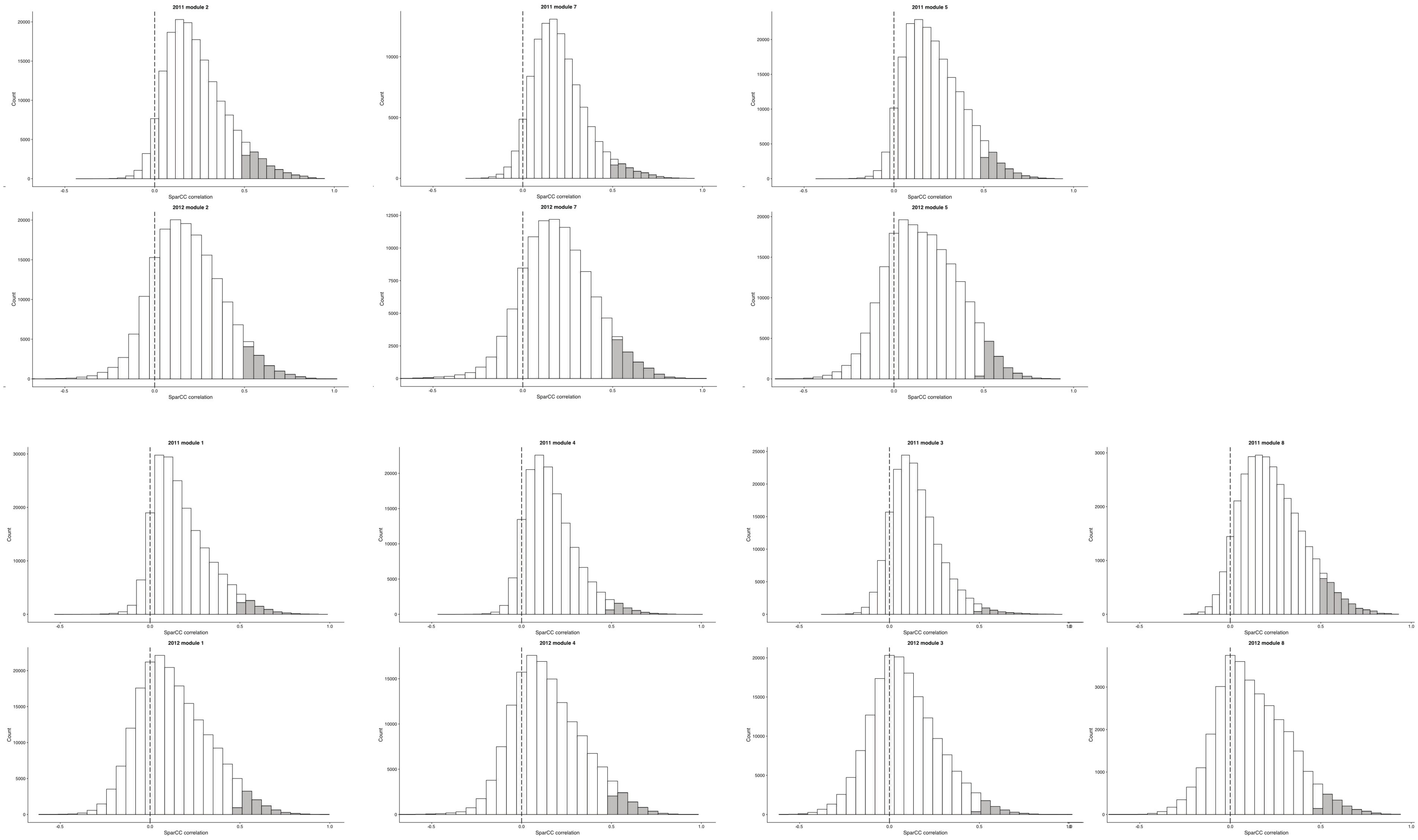

Figure S8. A majority of SparCC correlations within each module network are positive. Histograms of SparCC correlations are shown for each of the 2011 and 2012 consensus module networks. Dashed vertical lines mark a SparCC correlation of 0. Absolute correlation values >0.5 are shaded in grey. Most negative correlations are weak.
