# Supplementary material for: Recurrent patterns of microdiversity in a temperate coastal marine environment
Source: ISME J. 2017 Oct 24;12(1):237–52. doi: 10.1038/ismej.2017.165 (PMC5739018; doi:10.1038/ismej.2017.165)
Supplement: Supplementary Figure S9 [file ismej2017165x16.pdf]

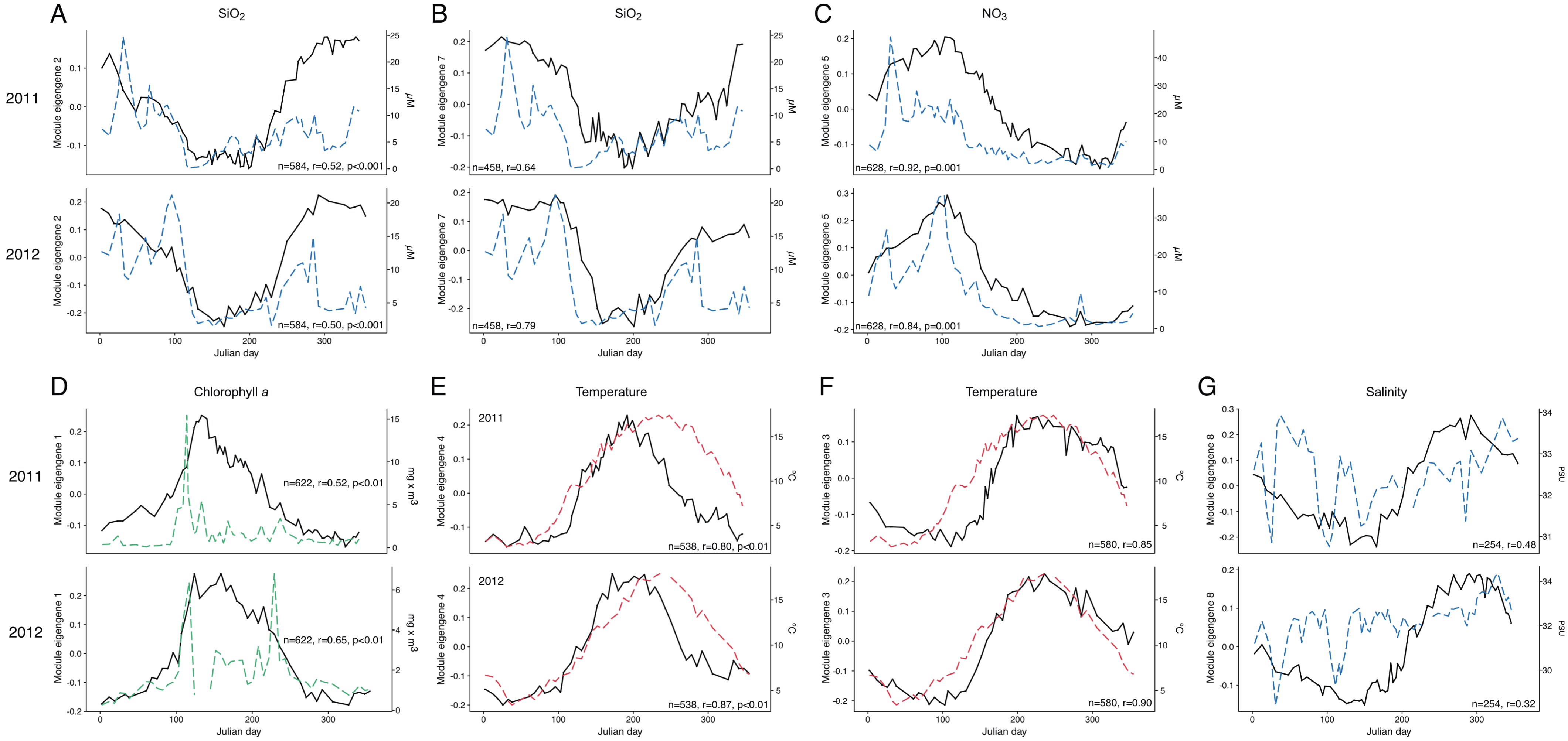

Figure S9. Module eigengenes (MEs) display recurrent correlations with environmental variables across 2011 and 2012. MEs (black lines) serve as a proxy for the collective abundance of its member oligotypes and are overlaid with their most strongly correlated environmental variable (dashed lines) (A-G) over Julian days represented on the x-axis. Inorganic nutrients are denoted blue dashed lines, chlorophyll *a* by green dashed lines and temperature by red dashed lines. ME trends are represented on the left y-axis and environmental variables on the right y-axis. n and r correspond to the total number of module oligotypes and Spearman correlation, respectively. *P*-values (p) are adjusted employing the method of Benjamini and Hochberg (1995). Correlations with inorganic nutrient SiO_2 is indicative of oligotrophic conditions when primary production is negligible and correlations with chlorophyll *a* are indicative of eutrophic conditions during phytoplankton blooms.
