# Supplementary material for: Recurrent patterns of microdiversity in a temperate coastal marine environment
Source: ISME J. 2017 Oct 24;12(1):237–52. doi: 10.1038/ismej.2017.165 (PMC5739018; doi:10.1038/ismej.2017.165)
Supplement: Supplementary Figure S10 [file ismej2017165x17.pdf]

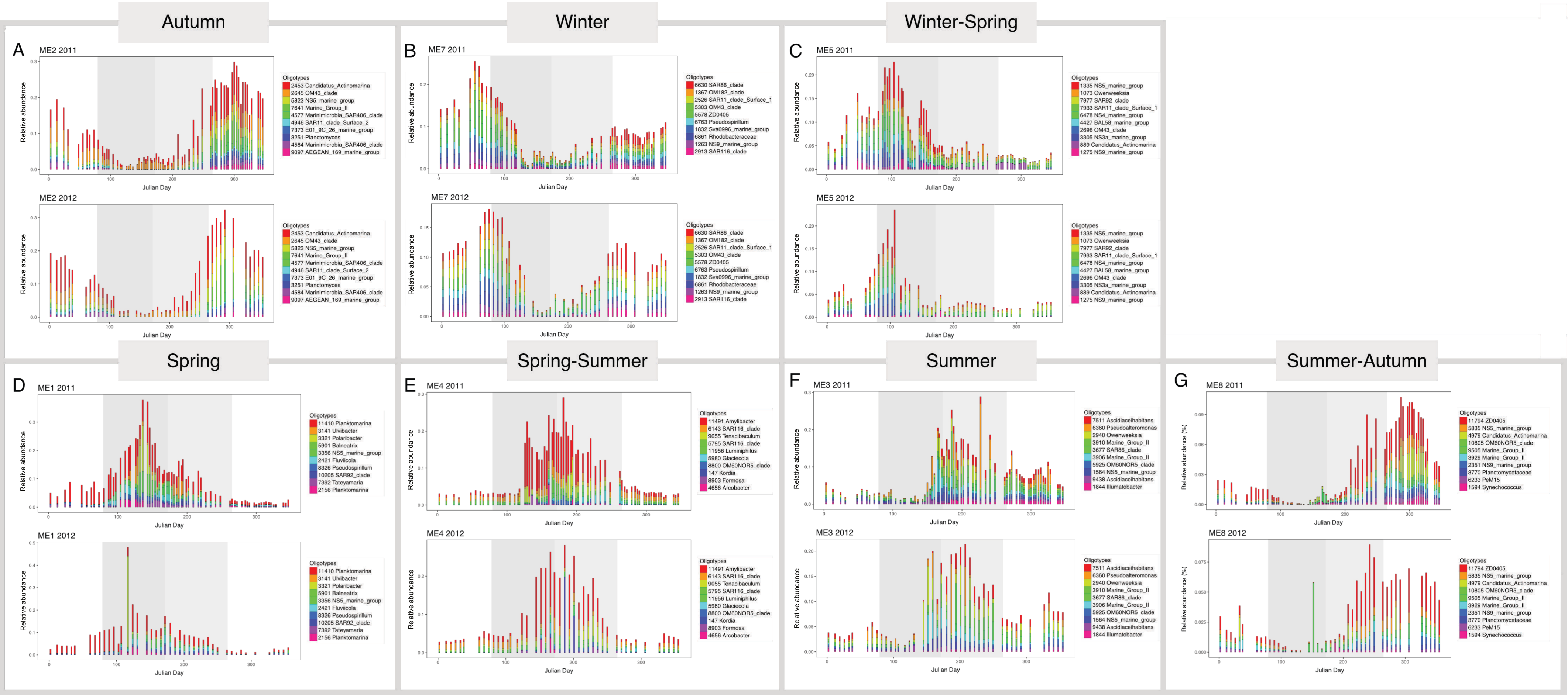

Figure S10. Module oligotype profiles reveal shifts in diversity patterns depending on the presence of bloom conditions. Relative abundance plots (A-G) display the top 10 most abundant module oligotypes. Modules are described according to their season of highest abundance: ‘autumn’ (A), ‘winter’ (B), ‘winter-spring’ (C), ‘spring’ (D), ‘spring-summer' (E), 'summer' (F) and 'summer-autumn'(G). Relative abundance is represented on the y-axis as a fraction and Julian days are represented on the x-axis. Spring and summer are denoted by the dark and light grey areas, respectively. Modules abundant in autumn and winter tend to display high and even diversity while modules abundant in spring and spring-summer display highly skewed diversity with a handful of oligotypes dominating relative community abundance.
