# Supplementary material for: Recurrent patterns of microdiversity in a temperate coastal marine environment
Source: ISME J. 2017 Oct 24;12(1):237–52. doi: 10.1038/ismej.2017.165 (PMC5739018; doi:10.1038/ismej.2017.165)
Supplement: Supplementary Figure S11 [file ismej2017165x18.pdf]

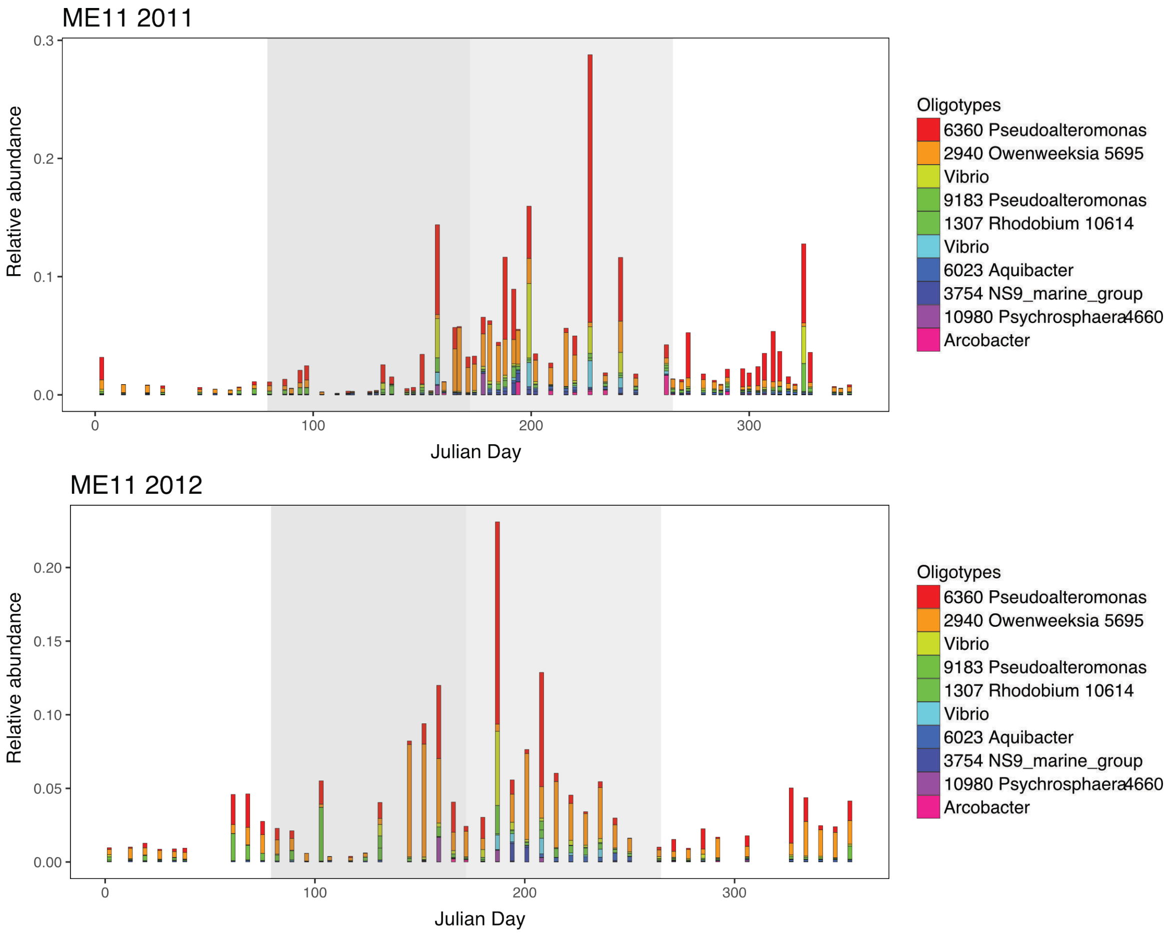

Figure S11. Un-merged module 11 harbors a collection of putative feast-and-famine oligotypes enriched in late spring and summer. Prior to merging with summer module 3, module 11 contained oligotypes with an abundance distribution distinct from all other un-merged modules where some taxa quickly reach high abundances followed by swift crashes particularly within *Gammaproteobacteria*. Julian days are shown on the x- axis and oligotype relative abundance as a fraction on the y-axis. Spring and summer are denoted by the dark and light grey areas, respectively. The top 10 most abundant module oligotypes are shown.
