# Supplementary material for: Recurrent patterns of microdiversity in a temperate coastal marine environment
Source: ISME J. 2017 Oct 24;12(1):237–52. doi: 10.1038/ismej.2017.165 (PMC5739018; doi:10.1038/ismej.2017.165)

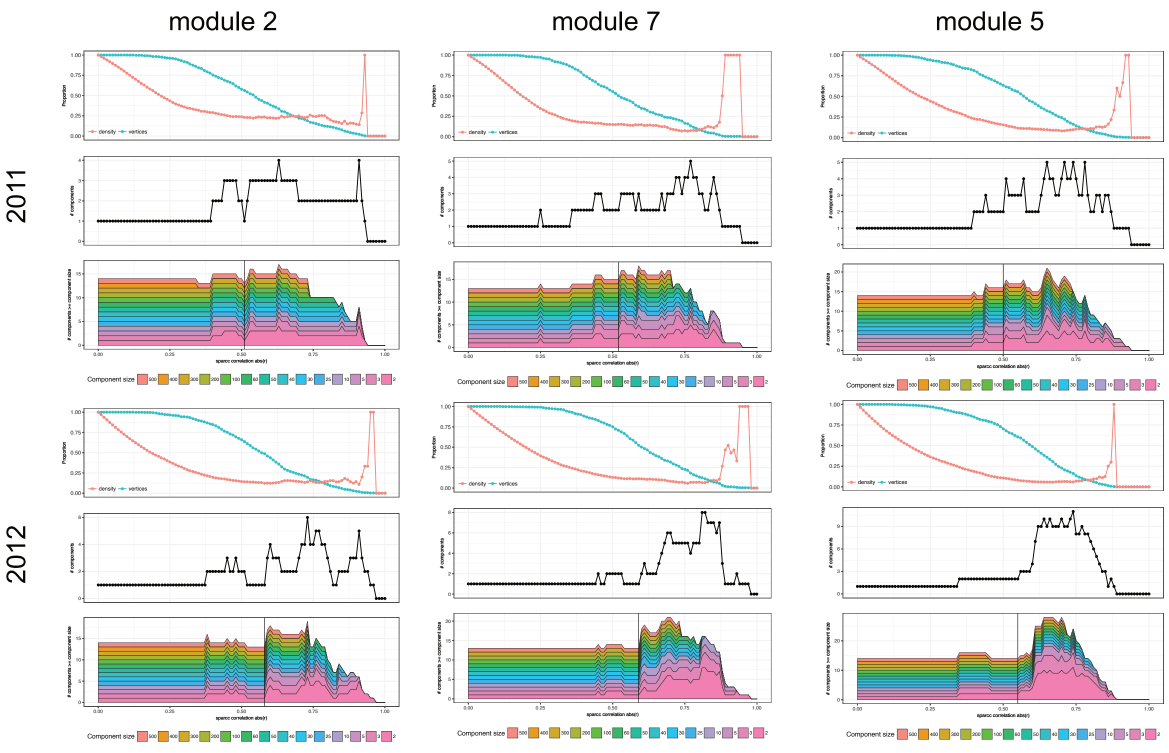


**Figure S12. The effect of SparCC correlation filtration thresholds on network graph characteristics for each module.** In order to maximize the edge filtration threshold that we could employ without breaking apart the entire module network, we tested a range of SparCC correlation thresholds (abs(r)) from 0 to 1 using igraph (Csardi and Nepusz, 2006). After edge filtration, vertices which became disconnected from all all other vertices (degree=0) were removed. For each threshold, we evaluated the effects on graph density and the proportion of vertices retained (top panels) and the total number of stand-alone sub-network components created as a result of filtration (middle panels). The bottom panels show the total number of components per size class after filtration (i.e., the total number of components that contain > 500, >400, >300, >100, >60, >50, >40, >30, >25, >10, >5, >3 and >2 vertices). The chosen filtration threshold is shown by the black line (Table 1). The schematic below illustrates the creation of individual sub-graph components after edge filtration. In this example, there are 2 components with >5 vertices and 1 component with >2 vertices after filtration.

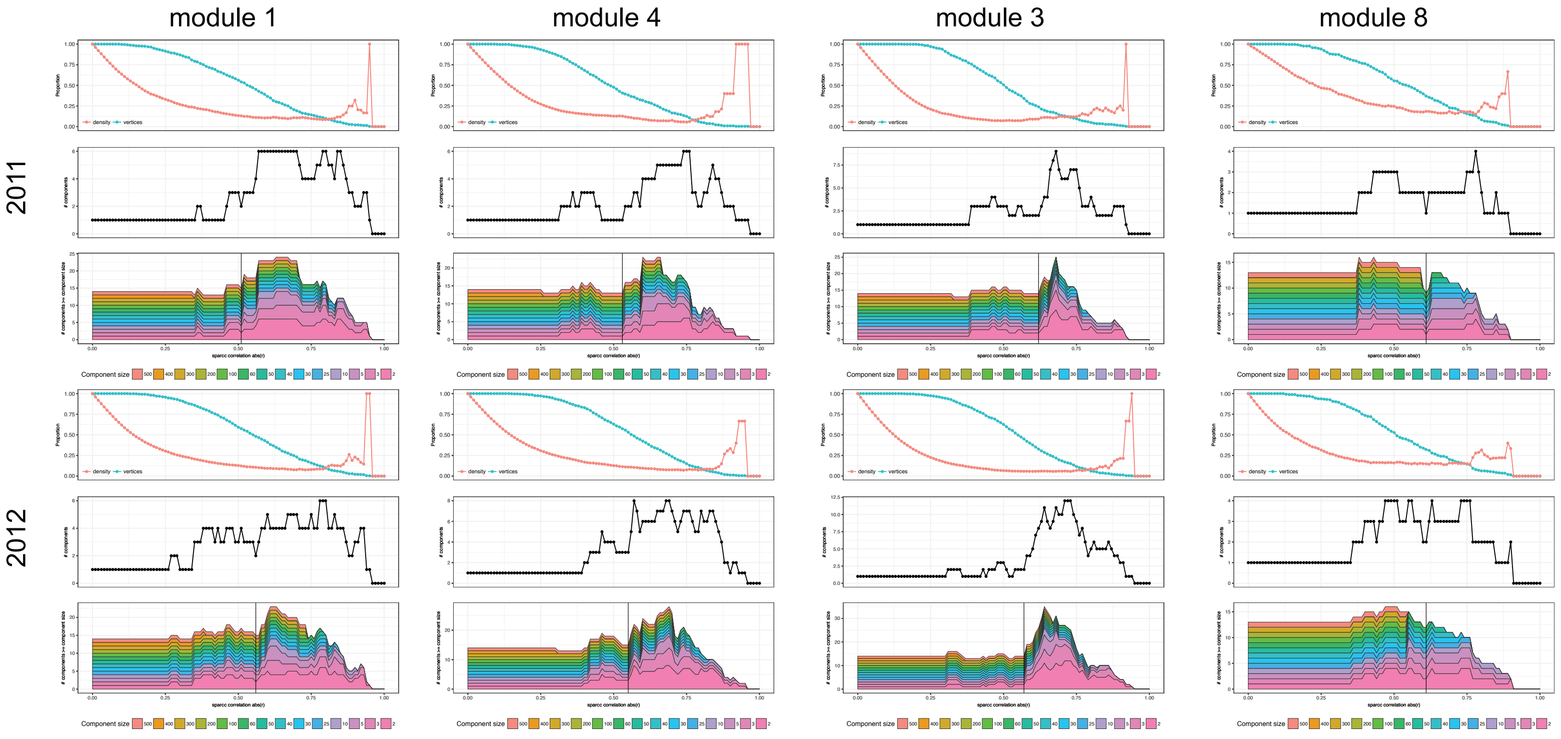


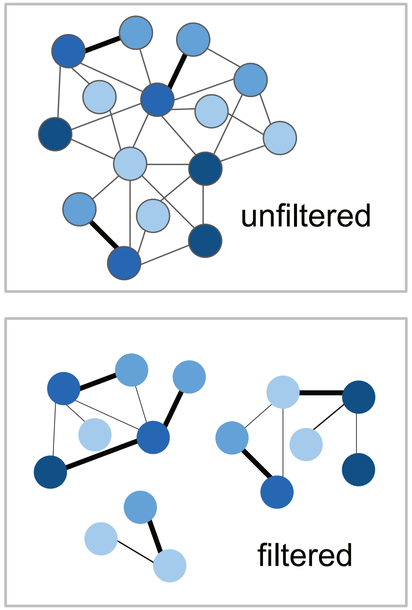

Supplement: Supplementary Figure S12 [file ismej2017165x19.pdf]
