# Supplementary material for: Recurrent patterns of microdiversity in a temperate coastal marine environment
Source: ISME J. 2017 Oct 24;12(1):237–52. doi: 10.1038/ismej.2017.165 (PMC5739018; doi:10.1038/ismej.2017.165)
Supplement: Supplementary Figure S13 [file ismej2017165x20.pdf]

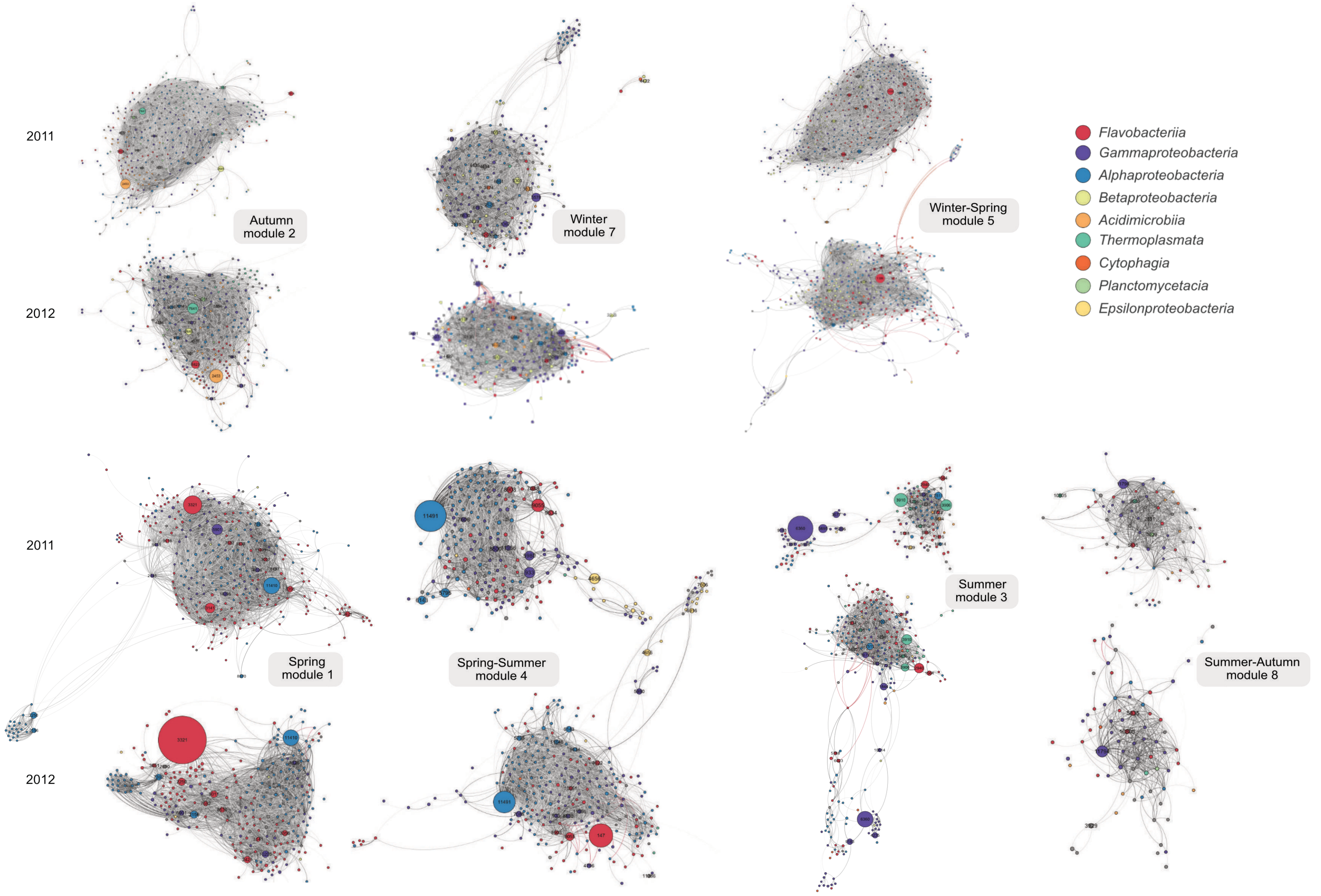

Figure S13. Individual filtered modules show similar networks of strongly correlated oligotypes across 2011 and 2012. Each module network was custom filtered to exclude edges with weak SparCC correlations (Table 1), followed by the removal of vertices which became disconnected from all other vertices (degree=0). Custom per-module filtration thresholds were employed to maintain the integrity of each main graph component (Figure S12). Vertex size is represented as the maximum observed oligotype abundance in 2011 and 2012 and is labeled with an oligotype id if abundance is >1%. Vertices are colored by class-level taxonomy. Edges are colored from light grey to black to denote weak to strong correlations. Decreases in community evenness can be seen in bloom compared to non-bloom seasons.
