# Supplementary material for: Recurrent patterns of microdiversity in a temperate coastal marine environment
Source: ISME J. 2017 Oct 24;12(1):237–52. doi: 10.1038/ismej.2017.165 (PMC5739018; doi:10.1038/ismej.2017.165)
Supplement: Supplementary Figure S14 [file ismej2017165x21.pdf]

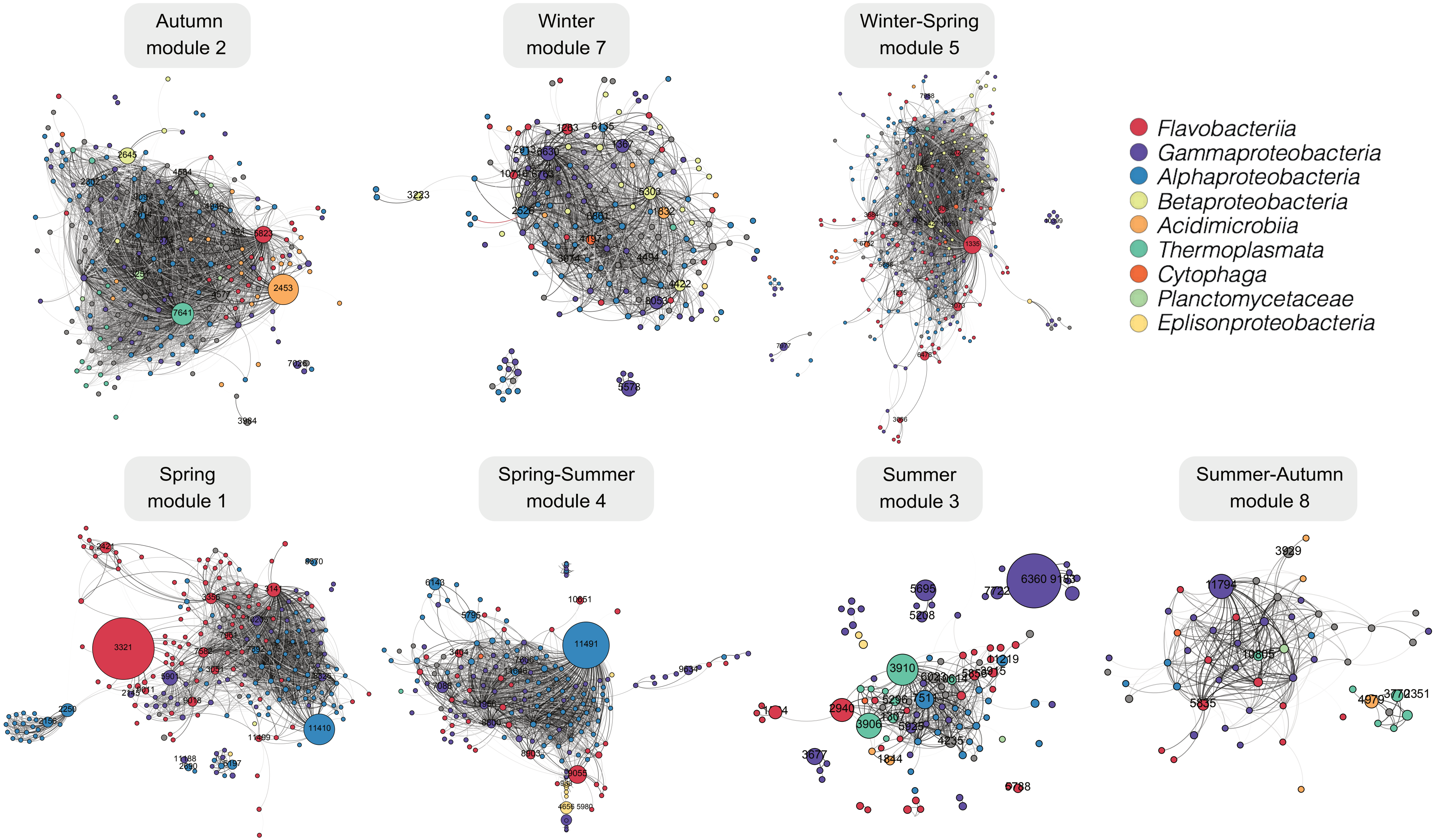

Figure S14. Intersected 2011 and 2012 module networks. Individual module networks from 2011 and 2012 were filtered to exclude edges with weak SparCC correlations (Table 1), followed by the removal of vertices which became disconnected from all other vertices (degree=0). Corresponding filtered module networks were then intersected using igraph (Csardi and Nepusz, 2006) to obtain single module representative of vertices and edges shared across 2011 and 2012 module networks. Between 53% and 75% of vertices (average 70%) were shared across both years, and between 32% and 49% of network edges (average 43%) were shared across both years (Table 1). Vertex size is represented as the average maximum observed oligotype abundance in 2011 and 2012 and is labeled with an oligotype id if its abundance is >1%. Vertices are colored by class-level taxonomy. Edges are colored from light grey to black to denote weak to strong correlations. Decreases in community evenness can be seen in bloom compared to non-bloom seasons.
