# Supplementary material for: Recurrent patterns of microdiversity in a temperate coastal marine environment
Source: ISME J. 2017 Oct 24;12(1):237–52. doi: 10.1038/ismej.2017.165 (PMC5739018; doi:10.1038/ismej.2017.165)
Supplement: Supplementary Figure S15 [file ismej2017165x22.pdf]

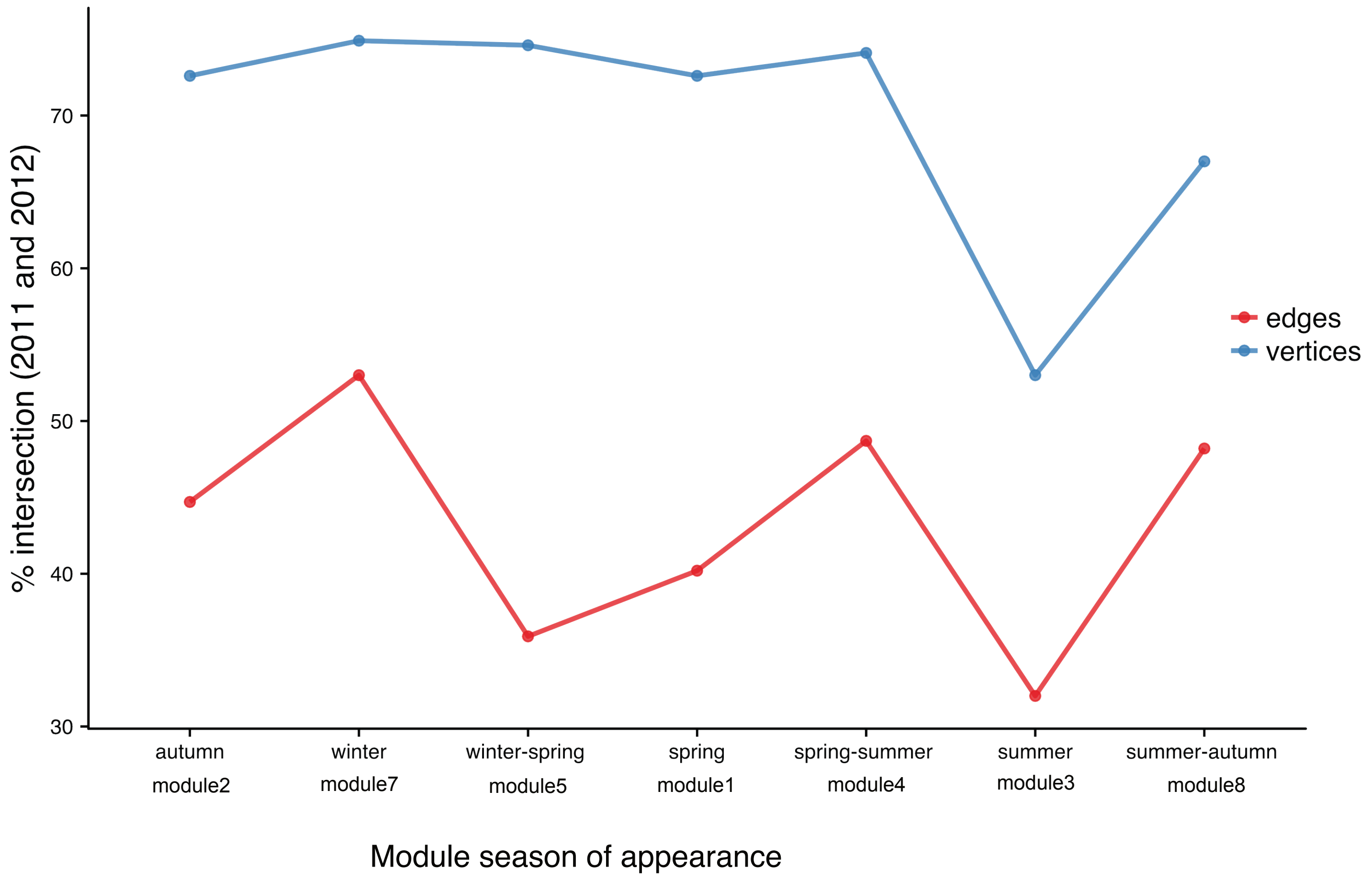

Figure S15. The proportion of shared edges and vertices across 2011 and 2012 module networks. Module networks were filtered to exclude weak SparCC correlations (Table 1), followed by the removal of vertices which became disconnected from all other vertices (degree=0). Corresponding filtered module networks were then intersected using igraph (Csardi and Nepusz, 2006) to obtain those vertices and edges conserved across 2011 and 2012 module networks. Between 53% and 75% of vertices (average 70%) were shared across both years, and between 32% and 53% of network edges (average 43%) were shared across both years (Table 1). Summer module 3 represents the period of lowest overlap in module networks with a decrease in both shared vertices and edges.
