# Supplementary material for: Recurrent patterns of microdiversity in a temperate coastal marine environment
Source: ISME J. 2017 Oct 24;12(1):237–52. doi: 10.1038/ismej.2017.165 (PMC5739018; doi:10.1038/ismej.2017.165)
Supplement: Supplementary Figure S16 [file ismej2017165x23.pdf]

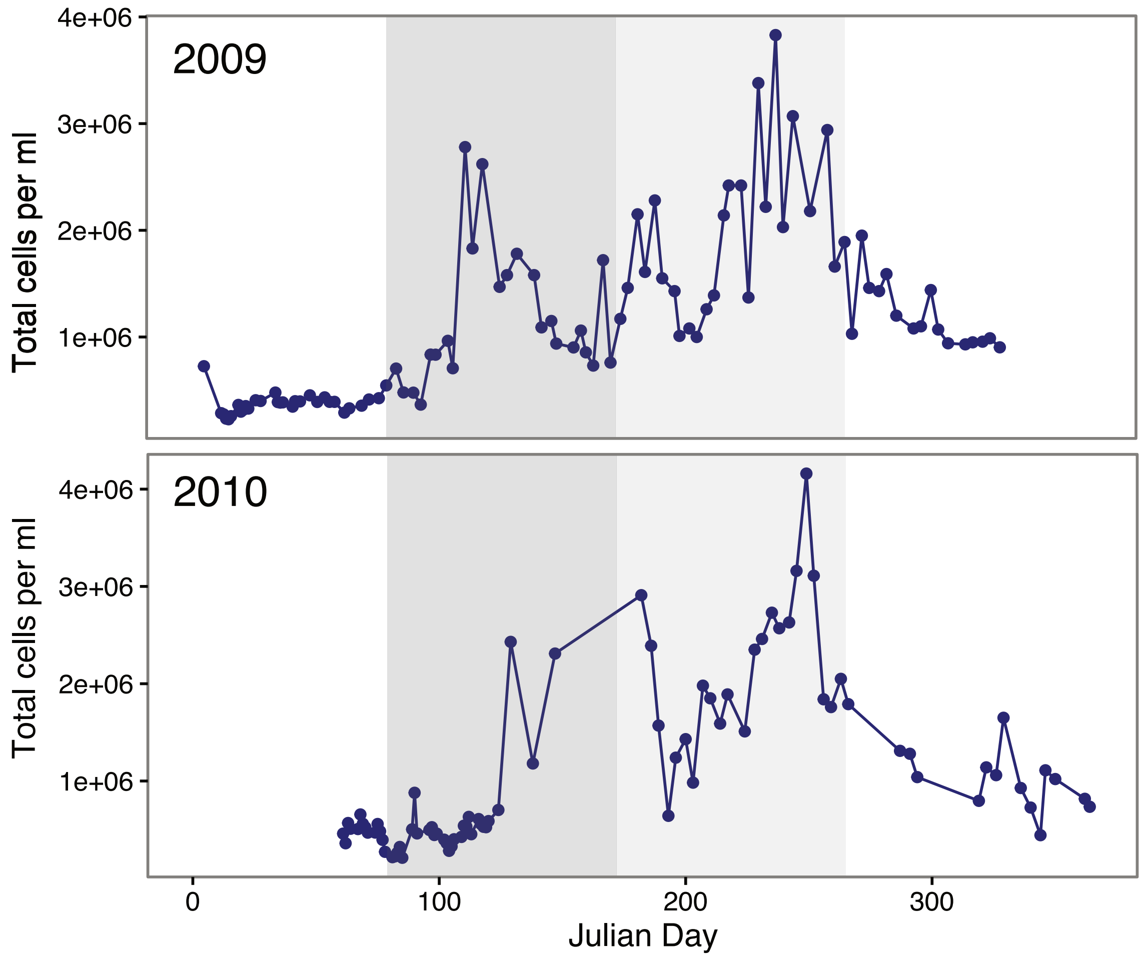

Figure S16. Bacterioplankton total cell counts (TCC) as a proxy for heterotrophic productivity. DAPI-based TCC from a previous study year (2009; Teeling et al, 2012) and 2010 are included here. Julian days are shown on the x-axis and cell counts per ml on the y-axis. Spring and summer are denoted by the dark and light grey areas, respectively. Average TCC per season include winter: 4.46e+5, spring: 8.78e+5, summer: 2.04e+6, autumn: 1.16e+6.
